# Adhesives and Sealants in Packaging: Functional Roles and System-Level Classification (Part I)

**DOI:** 10.3390/ma19112210

**Published:** 2026-05-24

**Authors:** Calogero Volpe, Leonardo Pagnotta

**Affiliations:** Department of Mechanical, Energy and Management Engineering, University of Calabria, Arcavacata, 87036 Rende, Italy; calogero.volpe@unical.it

**Keywords:** adhesives, sealants, interfacial adhesion, multilayer packaging, packaging materials, heat sealing, hot-melt adhesives, bio-based materials, recyclability, food packaging

## Abstract

Adhesives and sealants are critical yet still underrepresented components in packaging science. Existing reviews mainly address specific chemistries, sealing technologies, or application niches, whereas integrated analyses of adhesive and sealant families within a unified packaging-system framework remain limited. This review addresses this gap by proposing a three-dimensional classification framework—functional role, material chemistry and activation mechanism, and performance constraints—that connects functional roles, processing routes, regulatory constraints, and circularity requirements. The framework is applied across natural, synthetic, hot-melt, pressure-sensitive, and tie-layer adhesives, as well as conventional thermoplastic, barrier-oriented, and biodegradable sealant systems. Special attention is given to hybrid systems operating at the boundary between bonding and sealing, and to the performance–recyclability trade-offs that arise in multilayer architectures. Structure–property–function relationships are analysed qualitatively with respect to bond and seal strength, seal initiation temperature, hot-tack behaviour, and end-of-life compatibility. Part I establishes the classification and functional groundwork for the two-part review; Part II will extend the analysis to quantitative performance data, advanced materials, and emerging technologies.

## 1. Introduction

Adhesives and sealants constitute an indispensable yet often under-recognised class of materials in modern packaging systems. Their function extends well beyond simple joining, encompassing the mechanical integration of heterogeneous substrates, the continuity of barrier layers, the integrity of seals under thermal and mechanical loading, and the overall reliability of packaged products throughout storage, transport, and use [1,2]. Virtually all contemporary packaging formats—from rigid containers and closures to flexible laminates and multilayer films—depend on adhesive and sealing layers to achieve performance targets that cannot be met by monolithic materials alone [3,4].

Historically, packaging materials such as glass, metals, paper, and polymers have been discussed primarily in terms of bulk composition and intrinsic properties, while interfacial materials were treated as secondary or auxiliary components [5]. In practice, however, adhesives and sealants frequently represent the critical functional interface within packaging architectures. They govern stress transfer between layers, accommodate differential thermal expansion, enable high-speed converting and sealing operations, and often determine failure modes under mechanical abuse, thermal processing, or long-term storage conditions [3,6]. In multilayer systems, overall package performance is commonly limited not by the barrier or structural layer itself, but by the integrity and stability of the adhesive or sealant connecting adjacent materials [4].

This interface-centric role has become increasingly prominent as packaging systems have evolved toward higher complexity. Lightweighting strategies, the widespread adoption of multilayer and hybrid architectures, and the integration of active or functional elements have intensified reliance on tailored adhesive and sealing solutions [7]. In parallel, regulatory constraints related to food-contact safety, migration, and volatile emissions, together with growing emphasis on recyclability and circular design, have placed adhesives and sealants under heightened scrutiny [8,9]. Materials that were once largely invisible within packaging structures now emerge as key enablers—or potential bottlenecks—of sustainable packaging solutions [10].

Despite their central role, the literature addressing packaging adhesives and sealants remains fragmented. Many contributions focus on specific chemistries, application niches, or processing technologies, while broader reviews often treat adhesives and sealants separately, without explicitly addressing their functional overlap within integrated packaging systems. For instance, Ref. [11] provides a critical analysis of bio-based adhesive systems, highlighting both their functional potential and limitations in industrial implementation. Similarly, Ref. [12] offer a general overview of adhesives and sealants, discussing conventional and bio-based materials but without integrating bonding and sealing functions within packaging architectures. More specialised reviews, such as [13], focus on specific application domains (e.g., electronic packaging), further reflecting the sectoral fragmentation of the field.

As a result, a unified framework that simultaneously considers bonding and sealing functions, material chemistry, activation mechanisms, and system-level constraints in packaging applications is still lacking. The present review addresses this gap by adopting an integrated, system-oriented perspective. Comparative analyses linking material families, bonding or sealing mechanisms, functional performance metrics, regulatory requirements, and end-of-life implications are still limited. This fragmentation complicates material selection and hinders the development of coherent design strategies for advanced packaging applications.

**Figure 1:**
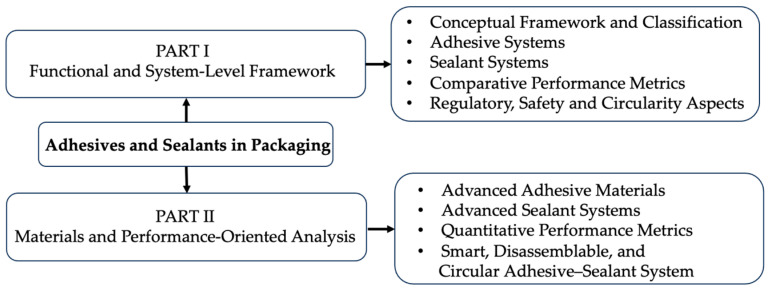
Roadmap of the two-part review on adhesives and sealants in packaging.

In this context, the present review adopts a system-level perspective, consistent with recent materials-oriented analyses of packaging glasses and metals [14,15]. Adhesives and sealants are examined not merely as chemical formulations, but as functional components embedded within packaging architectures. The review aims to connect material composition and interfacial mechanisms to processing routes, mechanical and barrier performance, regulatory compliance, and sustainability considerations, providing a unified framework for understanding and designing adhesive and sealing solutions in packaging. Figure 1 summarises the scope and structure of the two-part review and highlights the focus of Part I addressed in this work.

### 1.1. Historical Evolution of Adhesives and Sealants in Packaging

The use of adhesive and sealing materials long predates the emergence of modern industrial packaging systems. Archaeological and historical evidence indicates that natural binders such as bitumen, plant resins, waxes, starches, and protein-based glues were already employed in prehistoric and ancient societies to assemble composite artefacts, seal containers, and protect goods during storage and transport. These early materials fulfilled functions analogous to contemporary bonding and sealing by limiting leakage, stabilising closures, and providing mechanical cohesion, well before packaging was formalised as an industrial activity [16].

With the development of paper and paperboard packaging between the eighteenth and nineteenth centuries, adhesives assumed a more explicit structural role within packaging architectures. Starch- and dextrin-based formulations enabled the production of bags, cartons, and labels, supporting the expansion of industrial-scale distribution systems. Natural rubber cements and casein glues were progressively adopted for bonding composite paperboard structures, anticipating the multilayer concepts that would later become central to packaging design [3,4].

The twentieth century marked a decisive transition from predominantly natural formulations to synthetic adhesives and engineered sealants. Advances in polymer chemistry enabled the introduction of materials with improved bond strength, moisture resistance, and compatibility with metals and emerging polymer substrates. In parallel, the development of thermoplastic sealant layers—particularly polyolefin-based materials—established heat sealing as a distinct yet closely related functional domain, enabling the rapid growth of flexible packaging and high-speed converting operations [3,7].

From the late twentieth century onward, adhesives and sealants evolved from commodity materials into highly engineered components tailored to specific substrates, processes, and service conditions. Tie layers were developed to promote adhesion between chemically dissimilar polymers in co-extruded structures; pressure-sensitive adhesives enabled labelling and reclosable formats; and specialised sealants were designed to withstand retort, pasteurisation, and chemically aggressive environments. This period consolidated the role of interfacial materials as critical enablers of packaging functionality rather than ancillary components [4,7].

More recently, sustainability-driven considerations have reshaped the development of adhesive and sealing technologies. Requirements related to food-contact safety, solvent reduction, recyclability, and circular design have renewed interest in bio-based and hybrid formulations, not merely as direct substitutes for fossil-based systems, but as materials requiring dedicated redesign of formulations, processing conditions, and performance expectations [8,11]. In this context, adhesives and sealants increasingly act not only as performance enablers but also as potential constraints within circular packaging strategies.

Overall, the historical evolution of adhesives and sealants closely mirrors the broader transformation of packaging, from monolithic materials toward integrated, multilayer systems in which interfacial layers play a decisive role. Understanding this evolution provides essential context for contemporary adhesive and sealing technologies and for future developments aimed at balancing performance, safety, and sustainability in packaging systems [3,4].

### 1.2. Methodological Note

The reference base of this review was assembled through a structured literature search focused on adhesive and sealant technologies relevant to packaging systems. The survey drew on major scientific databases, including Scopus, Web of Science, ScienceDirect, and Google Scholar, complemented by publisher platforms and selected technical and regulatory sources relevant to packaging materials.

Search queries combined terms related to adhesive and sealant technologies, multilayer packaging structures, functional performance, food-contact safety, and recyclability. Representative search strings included combinations such as (adhesive OR sealant) AND packaging AND multilayer, complemented by terms related to sealing technologies, interfacial bonding, barrier performance, recyclability, and food-contact safety. Additional sources were identified through backward citation tracking of key review papers and technical reference works commonly used in the packaging literature.

The review was developed as a structured narrative synthesis rather than as a formal systematic review. Source selection prioritised relevance to packaging applications and excluded works focused exclusively on non-packaging sectors or lacking clear relevance to packaging performance. The literature survey primarily covered publications from 1995 to 2026, while earlier references were retained selectively when necessary to provide historical context or foundational classification schemes.

In total, 99 scientific and technical references were considered in this first part of the review, which focuses on classification criteria and functional roles. Additional references will be considered in Part II, which will extend the discussion to quantitative performance analysis and advanced material systems. In addition to these sources, the discussion refers, where relevant, to three key European regulatory frameworks governing packaging and food-contact materials, namely the Packaging and Packaging Waste Regulation (PPWR), Regulation (EC) No. 1935/2004, and Regulation (EC) No. 2023/2006.

**Figure 2:**
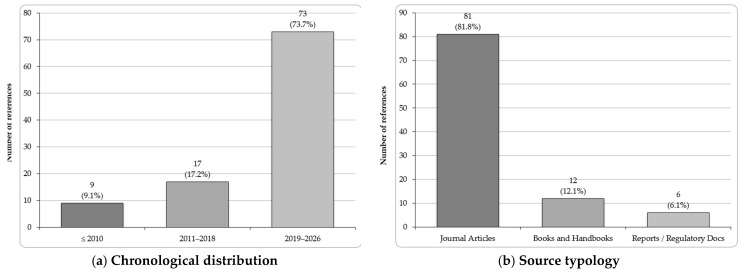
Overview of the documentary basis considered in this review. (**a**) Chronological distribution of the 99 scientific and technical references analysed in this study across three publication periods, highlighting the predominance of recent literature. (**b**) Distribution of the same sources by typology, including journal articles, books and handbooks and reports or technical documents.

The overall structure of the consulted literature is summarised in Figure 2. Figure 2a illustrates the chronological distribution of the references across three publication periods, highlighting the predominance of recent studies. Figure 2b presents the distribution of the same sources by typology, distinguishing journal articles, books and handbooks, and reports or technical documents.

This profile reflects a deliberate selection strategy. Earlier references were retained selectively for historical framing and foundational classification schemes, whereas the majority of the consulted literature was drawn from recent years to reflect current developments in packaging materials, interfacial technologies, and sustainability-driven design requirements.

Where performance metrics such as bond strength, seal strength, seal initiation temperature, and hot-tack are discussed qualitatively in this Part I, it should be noted that reported values in the literature are derived from a variety of test protocols—including ASTM F88 [17], DIN 55529 [18], and institution-specific methods—that differ in peel angle, seal bar geometry, specimen conditioning, and loading rate. Direct numerical comparisons across studies are therefore subject to methodological variability, which will be explicitly addressed in the quantitative analysis of Part II.

During the preparation of this manuscript, the authors used ChatGPT (GPT-4) to assist in drafting selected portions of the text and in generating some figures. All AI-assisted outputs were subsequently reviewed, revised, and validated by the authors to ensure scientific accuracy and consistency with the analytical framework of the review. The use of this tool did not involve the processing or analysis of primary research data, and full editorial responsibility for all content remains with the authors.

## 2. Conceptual Framework and Classification

Packaging adhesives and sealants are treated in this review as interfacial materials embedded in packaging architectures, whose performance cannot be evaluated independently from substrates, converting processes, and end-of-life constraints. Although the two classes are often discussed together, their distinction is primarily functional: adhesives provide structural bonding between layers or components, whereas sealants ensure hermetic closure and continuity of barrier performance at the package seam. Accordingly, adhesive and sealant denote functional categories rather than specific material classes, since similar polymer families may be formulated and processed to perform either bonding or sealing roles, and in some cases both, depending on activation conditions and application context [19,20]. This overlap is particularly relevant for hybrid systems, where the same formulation may contribute to structural coupling and closure integrity under different converting scenarios.

Sealing as a packaging function may also be achieved through monolithic materials such as glass and metals, which provide hermeticity via continuous structures, plastic deformation, or metallurgical joining. In this review, however, the term “sealant” is restricted to polymeric or composite interfacial layers applied to enable closure and barrier continuity.

A practical classification is therefore developed by integrating three complementary dimensions: (i) functional role within the packaging system, (ii) material chemistry and activation/processing mechanisms, and (iii) performance-related constraints tied to converting and in-use conditions. These dimensions are not mutually exclusive. In packaging systems, chemistry, activation route, application context, and performance requirements are strongly coupled; the framework is therefore intended as a multidimensional reading key that supports consistent comparison across technologies rather than as a set of independent, strictly separated taxonomies. Industrial classification manuals and packaging-focused reviews provide the conceptual basis for this organisation and are used here as a reference backbone to structure the subsequent sections [19,21,22].

While functional role and performance metrics represent individual classification dimensions, the chemistry and processing domain encompasses multiple interconnected criteria—including material origin, activation mechanism, and converting route—because these aspects are often coupled in practical packaging systems.

### 2.1. Role Within the Packaging System

Consistent with the classification axes introduced above, the first dimension is the functional role played by the interfacial material within the packaging architecture. In practice, adhesives are used to ensure structural coupling across components and layers, while sealants govern seam formation and barrier continuity at closures.

Within packaging architectures, adhesives contribute to system integrity through a limited set of recurring functions that can be described independently of the specific formulation. These functions include labeling (also in removable versions for reusable containers), lamination of multilayer films to combine barrier and mechanical functions, tie layers in coextruded structures to couple otherwise incompatible polymers, and special-function adhesives designed for conductive, reversible, or triggerable interfaces in smart and circular packaging concepts.

Sealants, by contrast, are responsible for hermetic closure and for limiting leakage and contamination. Their role is most critical in flexible and semi-rigid packaging, where seam integrity often constrains overall package performance. In this review, “sealants” refers to the polymeric (or composite) sealing-layer families that control heat-seal behaviour (or alternative sealing modes) under the combined constraints of barrier requirements, mechanical loading, and converting conditions.

Beyond these two primary functional categories, hybrid interfacial systems that blur the functional boundary between bonding and sealing, such as heat-sealable adhesives, hot-melt formulations used to support seam integrity, or debond-on-demand interfaces designed to enable delamination, are explicitly included because they directly influence recyclability and design-for-disassembly strategies.

### 2.2. Chemistry and Processing Mechanisms

Following the functional dimension discussed above, a second classification axis concerns material chemistry and activation or processing mechanisms, which govern interfacial formation, process integration, and compatibility with packaging substrates and converting operations. In packaging practice, these aspects are tightly linked to application feasibility and performance and are therefore discussed as part of an interdependent system perspective.

#### 2.2.1. Adhesives

To navigate the diversity of adhesive technologies used in packaging, three complementary criteria are adopted: chemical origin, bonding mechanism/processing route, and functional application context. These criteria are not intended as isolated “boxes”; they provide a practical reading key because, in packaging, composition, activation route, and application context typically constrain each other and jointly determine compliance and end-of-life compatibility.

- Chemical origin

Adhesives are broadly divided into natural systems [23] (e.g., starch, proteins such as casein/soy/gelatin, chitosan, lignin, tannins) and synthetic systems [2,6] (e.g., polyurethane, polyolefin-based, PVA, reactive systems tailored for strength and thermal behaviour).

- Bonding mechanism/processing route

These categories are distinguished according to the activation mechanism governing bond formation, which in packaging applications is closely linked to the corresponding processing route. Unlike chemical origin, which refers to material composition, this classification is based on how adhesion is activated and implemented during converting operations [19,24]. Because activation mechanisms typically determine the industrial processing route, bonding mechanisms and processing routes are treated jointly in this review.

The main mechanisms relevant to packaging include: reactive adhesives (chemical curing or crosslinking, typical of polyurethane systems); hot-melt adhesives (applied molten and solidifying on cooling, enabling high-throughput processing); pressure-sensitive adhesives (PSAs), which form bonds under light pressure at ambient temperature and are widely used in labeling and removable applications; water- or solvent-based systems, where adhesion develops through drying or evaporation processes, frequently used in paper-based packaging; and heat-sealable adhesives, thermally activated to enable closure of flexible or multilayer packaging structures [25,26,27].

- Functional application

Functional application is not treated as an independent classification dimension but as the operational expression of the functional roles introduced in Section 2.1. While *functional role* defines the purpose of the interface within the packaging architecture (structural coupling for adhesives), *functional application* identifies the specific design contexts in which that role is implemented and constrained by chemistry and converting route. Accordingly, labeling, lamination, tie-layer coupling, and special functions (including reversible/disassemblable interfaces) are treated as distinct design spaces because they impose different constraints on performance metrics, regulatory compliance, and end-of-life compatibility [3,28].

#### 2.2.2. Sealants

As for adhesives, sealants can be described through complementary classification dimensions including material chemistry, activation or sealing mechanism, and functional role within packaging systems. However, in sealant design the processing route and sealing mechanism often play a more dominant role, as they directly determine seam integrity, barrier continuity, and compatibility with converting conditions.

Sealants are grouped here into three packaging-relevant material classes, consistent with packaging-focused treatments:

- Conventional thermoplastic sealants, dominated by LDPE, PP, ethylene copolymers, and ionomers, selected for broad sealing windows and line compatibility [6,29].
- Multilayer barrier sealants, integrating high-barrier resins (e.g., EVOH, PVDC) and engineered structures to combine barrier protection with sealability [13,21].
- Biodegradable and bio-based sealants, including PLA, PBS, PBAT, protein/gelatin systems, waxes and starch derivatives, designed for compostable formats but often constrained by narrower sealing windows and moisture sensitivity [30].

Across these material classes, sealants may operate as permanent or peelable layers and may be activated by conventional heat sealing or alternative/non-thermal mechanisms depending on package design and converting constraints. Non-thermal or reversible sealing concepts are treated as an emerging sub-domain because they can reduce process energy and enable alternative end-of-life scenarios, particularly in high-performance multilayer structures.

### 2.3. Performance Metrics and System Constraints

Unlike the classifications in Section 2.1 and Section 2.2, which organise adhesives and sealants by functional role and by chemistry/activation route, this section adopts an operational classification based on measurable performance metrics and on system constraints imposed by converting lines and end-use conditions. In other words, materials are compared here according to how the interface behaves in service and during processing, rather than according to composition alone.

For adhesives, the key metrics include bonding strength (e.g., peel or shear strength), process window parameters (application temperature, open time, setting/curing time), substrate compatibility (wetting and adhesion on polymers, paper, metals, or coated surfaces), and safety constraints linked to migration, hazard profile, and food-contact compliance [31,32,33]. These metrics define whether a given adhesive family can be integrated into specific converting routes and whether performance remains stable under handling, storage, and transport conditions.

For sealants, performance is assessed through seam-related metrics, including seal strength, sealing temperature window, barrier continuity at the seam, and robustness against variability in pressure and dwell time on industrial equipment. In bio-based and moisture-sensitive systems, hot-tack behaviour and sealing consistency under humidity fluctuations become additional discriminators for line integration and process stability [7,21,34].

Finally, end-of-life compatibility is treated as a cross-cutting system constraint rather than as a standalone property. Multilayer performance gains can directly conflict with recyclability when interfaces cannot be separated or when incompatible chemistries contaminate recycling streams. This trade-off motivates the explicit consideration of innovation spaces such as debond-on-demand adhesives and reversible or lower-energy sealing concepts, which aim to reconcile interface performance during use with circularity requirements at end of life [8,10].

## 3. Adhesives in Packaging Architectures

Building on the three-dimensional framework introduced in Section 2, this section focuses on adhesive systems used in packaging and organises their classification primarily along the material and activation dimension, while explicitly maintaining links to functional roles and performance constraints. In this context, adhesives are treated as interface technologies embedded in packaging architectures, whose selection and classification are shaped by the coupled effects of functional role, processing route, substrate pairing, and end-of-life constraints, rather than by material chemistry alone. Sealants, which primarily govern closure integrity and barrier continuity at package seams, are discussed separately in Section 4.

**Figure 3:**
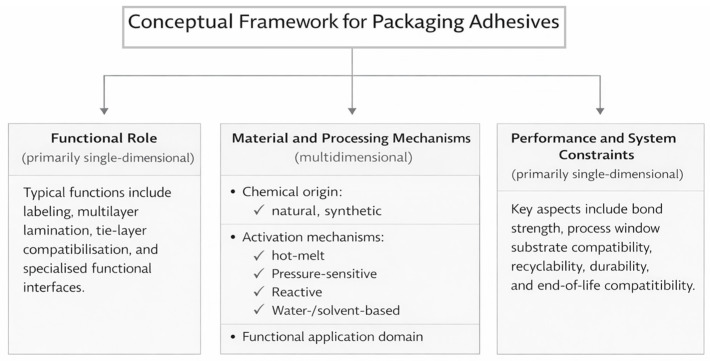
Conceptual framework for classifying packaging adhesives. Adhesive systems used in packaging are interpreted through three complementary dimensions: (i) functional role within packaging architectures (e.g., lamination, labeling, tie-layer coupling, specialised interfaces); (ii) material and processing mechanisms, including chemical origin and activation route; and (iii) performance and system constraints such as bond strength, processability, substrate compatibility, durability, recyclability, and end-of-life implications. The framework highlights how adhesive selection reflects a coupled optimisation between function, processing requirements, and packaging performance rather than a purely compositional classification.

As summarised in Figure 3, the discussion follows a system-oriented sequence. It first addresses what adhesives are required to accomplish within packaging architectures (e.g., labeling, lamination, tie-layer coupling, and special-function interfaces). It then examines how these interfaces are formed in practice through material chemistry and activation/processing mechanisms, where chemical origin, converting route, and application context are interdependent in industrial packaging lines. Finally, it considers the performance metrics and system constraints that determine feasibility and robustness, including bond strength, process window, substrate compatibility, durability, regulatory and migration requirements, and end-of-life implications. This organisation provides a consistent reading key for comparing adhesive technologies under packaging-relevant conditions, while maintaining alignment with the multidimensional framework defined in Section 2.

### 3.1. Functional Roles of Adhesives in Packaging Architectures

From a functional perspective, adhesives in packaging primarily ensure structural coupling between components and layers, contributing to mechanical integrity and system cohesion without directly governing closure hermeticity, which is instead the domain of sealants.

**Figure 4:**
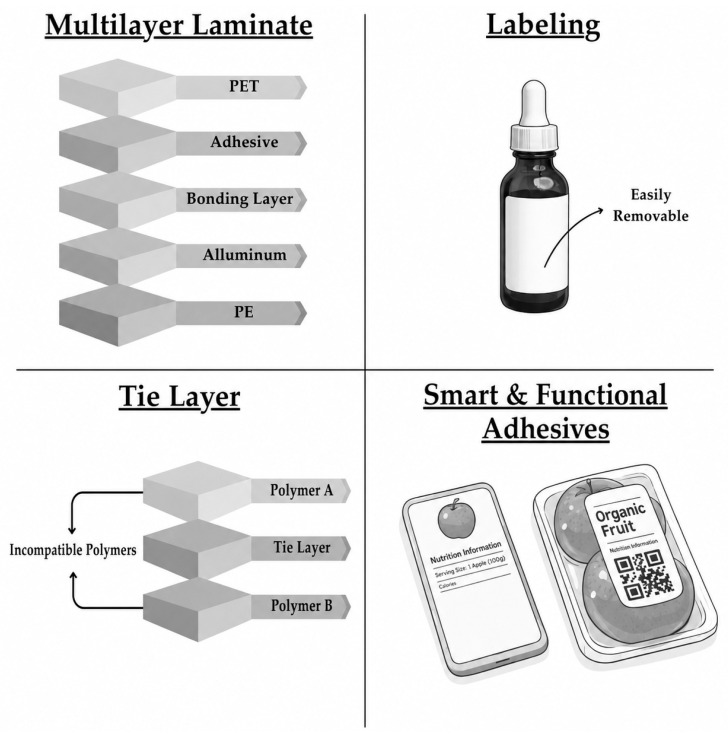
Functional roles of adhesives in packaging architectures. Schematic overview of the main functions performed by adhesives in packaging systems, including multilayer lamination, labeling applications, tie-layer coupling between incompatible materials, and emerging functional interfaces for smart or advanced packaging. The figure emphasises the role of adhesives as structural interfacial elements within packaging architectures rather than as closure-sealing materials.

The principal functional roles of adhesives in packaging architectures are summarised schematically in Figure 4, which highlights their contribution to multilayer lamination, labeling applications, compatibilising tie layers, and emerging smart or functional adhesive interfaces. This functional perspective provides a first, essentially monodimensional reading key that supports interpretation of adhesive selection before considering the more complex interplay of chemistry, processing conditions, and performance constraints addressed in subsequent sections.

A first major functional domain concerns multilayer lamination, widely used to combine complementary material properties such as mechanical strength, gas or moisture barrier performance, optical characteristics, and printability. Adhesives provide stress transfer across layers and maintain interfacial stability under mechanical loading, thermal cycling, and environmental exposure. In flexible and semi-rigid packaging, laminate integrity often depends more critically on adhesive performance than on the intrinsic strength of individual substrates [4,7].

Labeling represents another key functional application. Adhesives used for labels must balance sufficient bonding strength during distribution and use with controlled removability in recycling or reuse scenarios. Wash-off or removable adhesive systems are therefore increasingly relevant for containers designed for material recovery or repeated use, where adhesive residues may interfere with sorting operations and recycling efficiency [3,35].

**Figure 5:**
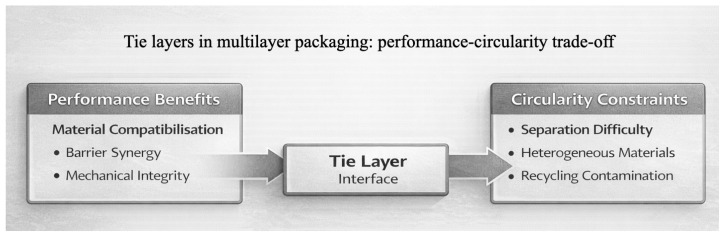
Tie layers in multilayer packaging: balancing performance and circularity. Tie layers act as compatibilising interfacial adhesives enabling adhesion between chemically dissimilar materials in multilayer packaging structures. While they enhance barrier performance, mechanical integrity, and material compatibility, they may introduce additional chemistries that complicate recycling, material separation, and end-of-life management.

Tie layers constitute a specialised functional adhesive role, particularly in coextruded or laminated structures where chemically incompatible polymers must be coupled. These interfacial systems enable multilayer architectures that would otherwise be unattainable due to differences in polarity, crystallinity, or surface energy between adjacent materials. Their primary function is not to maximise standalone bond strength but to ensure stress transfer and interfacial stability between layers characterised by mismatched physicochemical properties. While essential for achieving high-performance multilayer packaging, tie layers introduce additional interfacial chemistries that may complicate recycling and material separation at end of life. These performance–circularity trade-offs are illustrated schematically in Figure 5 [8,10].

Finally, specialised adhesive interfaces are emerging in advanced packaging concepts. These include conductive adhesives for smart packaging systems, reversible or debond-on-demand interfaces designed to facilitate disassembly and recycling, and functional adhesive layers contributing to sensing, active packaging, or intelligent labeling applications. Although still developing, these solutions illustrate how adhesive functionality increasingly extends beyond conventional structural bonding toward integrated packaging performance and system-level optimization [28,36].

Overall, while the functional classification of adhesives remains essentially monodimensional, it provides a useful starting point for interpreting material selection and packaging design strategies.

### 3.2. Material Chemistry and Processing Dimensions

While the functional perspective outlined in Section 3.1 provides a monodimensional interpretation of adhesive roles in packaging architectures, material selection and industrial implementation are inherently multidimensional. Adhesive performance cannot be interpreted solely in terms of chemical composition, as bonding behaviour, process integration, substrate compatibility, and end-of-life implications are strongly interdependent in practical packaging systems [19].

As introduced in the conceptual framework (Figure 3), three tightly coupled aspects govern adhesive technologies in packaging: chemical origin and formulation, activation mechanisms and processing routes, and the specific application context in which the adhesive interface operates. These dimensions rarely act independently. Chemical composition constrains activation mechanisms and processing conditions, while converting technologies and packaging design requirements, in turn, influence formulation choices and performance targets.

For clarity, this multidimensional domain is discussed through three complementary perspectives. The first concerns chemical origin, distinguishing broadly between natural or bio-based systems and synthetic polymer adhesives. The second addresses activation and processing mechanisms, including reactive curing systems, hot-melt technologies, pressure-sensitive adhesives, and water- or solvent-based dispersions, whose processing behaviour strongly affects industrial feasibility. The third considers application-driven formulation, where adhesive design reflects specific packaging functions such as multilayer lamination, labeling, tie-layer coupling, or specialised functional interfaces.

This organisation does not represent separate taxonomic classes but rather an operational framework that facilitates comparison across adhesive technologies while maintaining alignment with packaging performance, processing constraints, and circularity objectives.

#### 3.2.1. Chemical Origin

From the perspective of material origin, adhesive systems used in packaging can be broadly divided into two main categories: natural or bio-based adhesives derived from renewable resources, and synthetic polymer adhesives engineered primarily from petrochemical feedstocks. Although this distinction represents a simplified classification, it provides a useful starting point for understanding how formulation strategies reflect performance requirements, processing constraints, and increasingly, sustainability considerations in packaging design.

Natural and bio-based adhesives include systems derived from polysaccharides, proteins, lignin derivatives, and other bio-sourced components. Historically, these materials constituted the earliest bonding technologies in packaging and remain widely used in fibre-based applications such as paperboard lamination, labeling, and corrugated packaging. Their appeal lies in renewable sourcing, potential biodegradability, and compatibility with recycling streams, particularly in paper-dominated packaging formats. However, their performance is often sensitive to moisture, thermal fluctuations, and long-term environmental exposure, which can limit their applicability in high-barrier or mechanically demanding packaging structures. Ongoing research therefore focuses on modification strategies such as crosslinking, blending, or hybridisation with synthetic components to enhance durability while preserving sustainability advantages [23,37].

Synthetic adhesives, by contrast, dominate modern packaging applications where high mechanical performance, chemical resistance, and process robustness are required. These systems encompass a wide range of polymer chemistries, including vinyl polymers, polyurethanes, elastomeric formulations, reactive resins, and specialised functional polymers designed for multilayer lamination and structural bonding. Their versatility enables reliable adhesion across dissimilar substrates such as plastics, metals, coated papers, and composite materials, supporting high-speed industrial converting operations. At the same time, their chemical complexity and strong interfacial bonding may complicate recycling processes, particularly in multilayer packaging where adhesive layers hinder material separation [2,6].

Despite the apparent contrast between natural and synthetic systems, current developments increasingly blur this distinction. Hybrid formulations, bio-attributed polymers, and partially renewable synthetic adhesives are being explored to reconcile performance requirements with circularity objectives [38]. Consequently, chemical origin should be seen not as a rigid classification but as one factor within the broader decision-making framework for adhesive selection in packaging.

In packaging practice, natural and bio-based adhesives are predominantly associated with fibre-based substrates—paper, paperboard, and corrugated board—where surface porosity and chemical affinity facilitate adhesion and recycling compatibility [20,29], while synthetic systems are required for bonding chemically heterogeneous substrates including polyolefin films, aluminum foils, metallised layers, and coated barrier materials typical of flexible and multilayer packaging [21,39].

#### 3.2.2. Natural and Bio-Based Adhesives

Within the chemical-origin dimension outlined above, natural and bio-based adhesives represent one of the two main formulation domains in packaging applications.

Natural adhesives derive from renewable resources and are widely appreciated for their sustainability, biodegradability, and compatibility with environmentally friendly packaging systems. These adhesive systems represent the historical foundation of bonding technologies in packaging and have regained interest in response to sustainability-driven design strategies [4,7,40].

These systems are primarily derived from polysaccharides (starch, dextrin, cellulose derivatives), proteins (casein, gelatin, soy), and other bio-sourced components such as lignin, tannins, or chitosan [23]. They are preferentially associated with paper- and fibre-based substrates, where surface porosity and chemical affinity facilitate adhesion.

**Protein-based** formulations, typically derived from casein, soy proteins, or gelatin, represent a mature and well-established subgroup. Casein-based adhesives, obtained from milk proteins, constitute a valuable alternative to synthetic systems, especially for labeling applications on reusable containers such as glass and PET. Their main advantage lies in their removability during industrial washing processes, which facilitates the recycling of packaging components [35].

**Polysaccharide-based** adhesives include materials such as starch, cellulose derivatives, and chitosan. These systems are especially common in the paper and cardboard industry due to their low cost, biodegradability, and ease of application—typically in aqueous solution form, without the need for organic solvents [29]. However, their adhesion performance is sensitive to moisture, requiring careful control of humidity during use.

From a functional standpoint, bio-based adhesives are mainly employed in labeling, paperboard lamination, and corrugated packaging, where moderate bond strength, low processing temperatures, and compatibility with aqueous processing routes are advantageous [3]. Their bonding behaviour is dominated by hydrogen bonding and physical entanglement, with limited covalent crosslinking, which constrains resistance to moisture, thermal cycling, and mechanical stress and limits their applicability in high-barrier or high-performance packaging formats [37].

**Figure 6:**
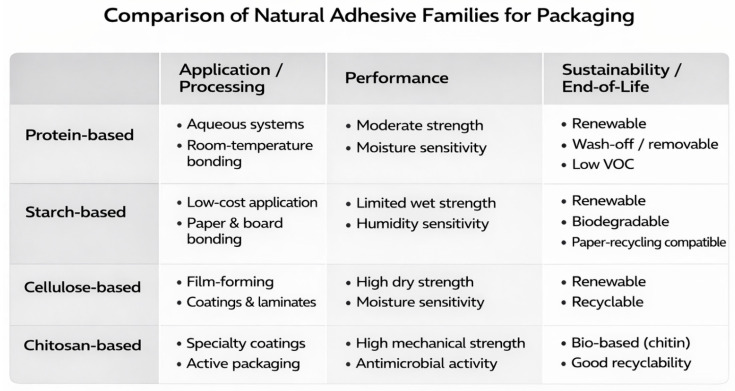
Comparison of natural adhesive families for packaging applications. Comparative overview of the main families of natural adhesives used in packaging—protein-based, starch-based, cellulose-based, and chitosan-based—organized according to three packaging-relevant dimensions: application and processing characteristics, performance attributes, and sustainability/end-of-life aspects. The figure highlights typical advantages and limitations of each family in relation to bonding behaviour, moisture sensitivity, and compatibility with recyclable and bio-based packaging systems.

Recent developments in the packaging literature emphasise modification strategies aimed at improving water resistance, setting time, and process robustness, including blending, crosslinking, and hybridisation with synthetic components. In parallel, the relevance of bio-based adhesives has expanded in the context of recyclability and compostability, where chemical compatibility with paper recycling streams and biodegradable substrates reduces contamination at end of life [8]. Figure 6 shows the main classes of natural adhesives, their application process, performance, and considerations regarding their sustainability and end-of-life.

#### 3.2.3. Synthetic Adhesives

Complementary to natural systems, synthetic adhesives constitute the second major formulation domain within the chemical-origin classification relevant to packaging applications.

Synthetic adhesives represent the dominant bonding technologies in modern packaging, owing to their broad formulation space, high mechanical performance, and robustness under demanding processing and service conditions [2,3]. Unlike natural systems, synthetic adhesives are primarily engineered to deliver consistent adhesion across chemically dissimilar substrates, high-speed converting compatibility, and resistance to moisture, temperature fluctuations, and mechanical stresses typical of industrial packaging environments.

From a materials perspective, the main synthetic adhesive families used in packaging include polyvinyl acetate (PVAc) and polyvinyl alcohol (PVA) dispersions, polyurethane-based systems, elastomeric formulations, and reactive or specialty adhesives tailored for structural bonding and multilayer architectures [6]. These systems are predominantly derived from petroleum-based polymers and encompass both thermoplastic and thermosetting chemistries, enabling fine control over rheology, curing behaviour, and final bond properties.

Within packaging architectures, synthetic adhesives are widely employed in multilayer lamination, structural bonding of composite components, and high-performance labeling, where strong and durable adhesion to polymers, metals, paper, and coated surfaces is required [4]. Their formulations are compatible with a variety of application routes, including water-based dispersions, solvent-based systems, hot-melt technologies, and solvent-free reactive formulations, allowing integration into high-speed industrial lines with tight process windows.

Compared with natural adhesives, synthetic systems exhibit superior bonding strength, enhanced resistance to moisture and thermal cycling, and improved long-term mechanical stability. These advantages arise from bonding mechanisms that combine physical interactions with chemical crosslinking or curing reactions. Polyurethane-based adhesives, particularly two-component (2K) systems, constitute a key class in this respect and are extensively used in flexible and multilayer food packaging, where they provide high bond strength and flexibility while preserving laminate integrity under demanding converting and service conditions [21].

Despite their performance advantages, synthetic adhesives pose significant challenges in terms of recyclability and circularity. Their chemical complexity, persistence, and strong interfacial bonding can hinder mechanical recycling by contaminating polymer streams or preventing efficient layer separation in multilayer structures [8,41]. These limitations highlight a fundamental performance–circularity trade-off in packaging design, motivating current research efforts toward solvent-free formulations, reduced-VOC systems, and debond-on-demand or compatibilizing strategies [38].

**Figure 7:**
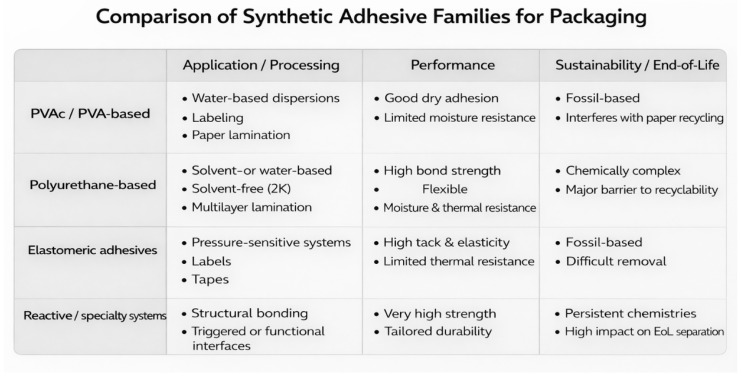
Comparison of the main synthetic adhesive families used in packaging, including PVAc/PVA-based systems, polyurethane-based adhesives, elastomeric adhesives, and reactive or specialty systems. The comparison highlights typical application and processing routes, key performance attributes, and sustainability and end-of-life implications.

The main characteristics of synthetic adhesive families, in terms of application, performance, and end-of-life implications, are summarized schematically in Figure 7.

A comparative reading of natural/bio-based and synthetic adhesive systems across the dimensions most relevant to packaging selection reveals a set of consistent and structurally opposed trade-offs. In terms of functional performance, synthetic systems—particularly polyurethane-based and reactive formulations—provide superior bond strength, resistance to moisture and thermal cycling, and compatibility with high-speed converting lines, while bio-based systems are generally constrained to moderate bond strength, narrower processing windows, and greater sensitivity to humidity and temperature fluctuations, limiting their applicability in high-barrier or mechanically demanding packaging formats [21,37]. From a cost perspective, bio-based adhesives derived from starch, proteins, or cellulose derivatives typically offer lower raw material costs and simpler aqueous processing routes, particularly in fibre-based packaging, whereas high-performance synthetic systems—especially two-component polyurethane laminates—involve higher formulation and processing costs but deliver performance levels unattainable with natural systems [2,11,29]. Regarding end-of-life compatibility, bio-based systems are generally more compatible with paper recycling streams and compostable formats, while synthetic adhesives may introduce contamination risks or hinder material separation in multilayer structures [8,38,42]. However, the circularity advantage of bio-based adhesives remains system-dependent: it is most evident in mono-material fibre-based packaging, whereas in multilayer structures even bio-based adhesive interfaces can hinder delamination if they maintain excessive interfacial strength during mechanical recycling. Modification strategies—including crosslinking of starch- and protein-based matrices, blending or hybridisation with synthetic components, nanostructured reinforcement approaches, and bio-attribution of synthetic polymer backbones—are progressively narrowing the performance gap, although industrially validated bio-based alternatives to synthetic laminates in flexible food packaging remain limited.

Among currently explored systems, protein-based adhesives and modified polysaccharide formulations appear the most mature for fibre-based food packaging applications, whereas bio-attributed polyurethane systems presently represent the most industrially vi-able approach for multilayer flexible packaging. Fully bio-based alternatives capable of re-placing conventional polyurethane laminates under demanding industrial converting conditions remain limited.

Quantitative benchmarking of this convergence, including performance data under standardised test protocols, is addressed in Part II of this review.

### 3.3. Activation Mechanisms and Processing Routes

Beyond chemical origin, adhesive technologies in packaging are strongly characterised by the mechanisms through which bonding is activated and implemented during converting operations. From the perspective of the three-dimensional framework, these activation mechanisms represent the material/processing dimension, but their relevance in packaging derives from their coupling with functional roles and system-level constraints such as line speed, substrate compatibility, durability, migration control, and end-of-life compatibility. In practical packaging systems, activation route and processing conditions often play a more decisive role than chemistry alone, as they directly affect line integration, production speed, substrate compatibility, and operational safety [24].

Adhesive bonding in packaging may be achieved through several activation mechanisms, including chemical curing reactions, thermal softening and solidification, pressure-induced adhesion, or solvent/water evaporation processes [19,28]. These mechanisms are closely linked to industrial processing routes and therefore influence formulation design, equipment requirements, and achievable performance levels.

Because activation mechanism, converting technology, and functional application context are tightly coupled in packaging practice, they are considered jointly in this review. This perspective allows adhesive systems to be interpreted not simply as material classes but as process-integrated interface technologies whose performance depends on the interaction between formulation, processing conditions, and packaging architecture.

The main activation routes relevant to packaging applications—including hot-melt systems, pressure-sensitive adhesives, reactive adhesives, and dispersion-based technologies—are discussed below in relation to their processing characteristics, application domains, and implications for packaging performance and circularity.

#### 3.3.1. Hot-Melt Adhesives

Within the activation-mechanism perspective introduced in Section 3.2.2, hot-melt adhesives are thermoplastic systems applied in the molten state (typically 120–180 °C) and solidifying upon cooling, enabling rapid setting and immediate handling. Their solvent-free nature and short setting times make them particularly compatible with high-throughput packaging lines, where process speed, clean operation, and tight converting windows are central requirements [1,6,25]. In packaging practice, hot-melt adhesives are widely used in carton sealing, case and tray forming, labeling, and selected lamination operations, where fast bonding and good cohesive strength after solidification are required [2]. Their performance is governed by viscoelastic behaviour, which must be balanced to ensure adequate wetting during application and sufficient cohesion after cooling. Because bonding develops without curing reactions, process implementation is simplified; however, thermal resistance is intrinsically limited compared with reactive systems, particularly for packages exposed to elevated temperatures during storage, transport, or use. Operational safety constraints related to molten application temperatures also remain relevant in industrial settings [25,43].

**Figure 8:**
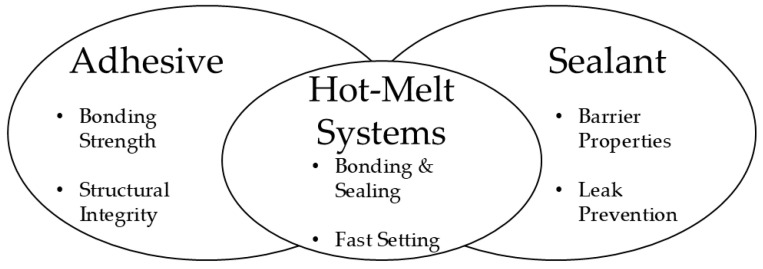
Positioning of Hot-Melt Systems. Dual role of Hot-Melt Systems. They serve both to join different components and to ensure structural continuity and prevent leakage.

A distinctive aspect in packaging architectures is the functional overlap between bonding and sealing when hot-melt formulations are used to support seam integrity or closure continuity. In such cases, the same material may contribute to structural coupling and to leakage prevention depending on the package design and the converting scenario, motivating their positioning at the interface between adhesive and sealant functionalities (Figure 8). This dual role can simplify packaging architectures but may also introduce constraints related to migration control, thermal stability, and compatibility with recycling operations [8,9,44].

Accordingly, ongoing developments increasingly focus on solvent-free and lower-impact formulations, including hybrid systems and bio-attributed chemistries, aiming to preserve process efficiency and bonding performance while improving sustainability and end-of-life compatibility [22,45]. Overall, hot-melt selection in packaging reflects the coupled requirements of converting speed, substrate wetting and cohesion, functional role within the architecture, and circularity constraints.

#### 3.3.2. Pressure-Sensitive Adhesives

Within the activation-mechanism dimension of packaging adhesives, PSAs represent systems that form bonds under light pressure at ambient temperature without requiring heat, solvents, or curing reactions. Adhesion develops through controlled viscoelastic behaviour, enabling immediate bonding while maintaining sufficient cohesion to resist detachment during handling, transport, and use [25]. In packaging, PSAs are predominantly associated with labels, tapes, and reclosable features, where ease of application and removability are critical [26].

The functional performance of PSAs relies on a controlled balance between adhesion, cohesion, and viscoelastic dissipation. In packaging contexts, this balance must be maintained across a wide range of temperatures, humidity levels, and surface conditions, particularly for containers subjected to refrigeration, condensation, or mechanical handling. For reusable or refillable packaging, PSAs must additionally allow clean removal without residue, imposing further constraints on formulation and surface interaction [46].

**Figure 9:**
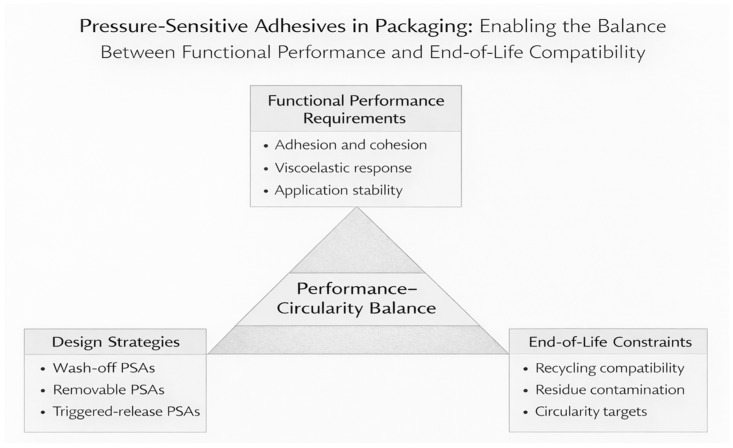
Pressure-sensitive adhesives in packaging: balancing functional performance and end-of-life compatibility. Functional performance requirements, end-of-life constraints, and design strategies jointly determine the optimisation of pressure-sensitive adhesive systems in packaging applications. Tailored PSA formulations enable reliable adhesion during use while facilitating clean removability and minimising contamination of recycling streams, thereby supporting circular packaging design.

From a circularity perspective, PSAs occupy a strategic position at the interface between functional performance and end-of-life compatibility. Adhesive residues from labels are a recognised source of contamination in glass and polymer recycling streams, prompting the development of wash-off, removable, and triggered-release PSA systems designed to facilitate separation during recycling operations [8,10]. Rather than representing a simple functional category, PSAs therefore illustrate how adhesive design can actively mediate the balance between packaging performance requirements and circularity objectives, as schematically summarised in Figure 9.

#### 3.3.3. Reactive Adhesives

Within the activation-mechanism classification, reactive adhesives comprise systems in which bonding develops through chemical curing or crosslinking reactions occurring during or after application [47]. These formulations are widely employed in packaging where high mechanical strength, chemical resistance, and long-term stability under demanding service conditions are required.

Reactive polyurethane-based adhesives represent the most established class in flexible and multilayer packaging, particularly for food-contact laminates combining polymers, aluminum foils, coated papers, or composite barrier structures [3,6,21]. In these systems, curing reactions typically occur between functional polymer components or through moisture-triggered crosslinking, generating cohesive networks that provide strong interfacial adhesion and resistance to thermal, mechanical, and environmental stresses.

Compared with thermoplastic adhesive systems, reactive adhesives generally offer superior thermal stability, chemical resistance, and durability [25]. These properties are particularly relevant in multilayer flexible packaging, where laminate integrity must be maintained during converting operations, storage, transport, and use. Their ability to bond chemically dissimilar substrates also makes them key enabling technologies for high-barrier packaging architectures [48].

However, reactive adhesive systems introduce specific processing and end-of-life challenges. Curing kinetics, residual monomer control, and migration compliance must be carefully managed, especially in food packaging applications [44]. Additionally, the strong interfacial bonding and chemical complexity of cured adhesive layers can hinder material separation and recycling in multilayer packaging structures, contributing to the well-known performance–circularity trade-off in advanced packaging systems [8].

Current developments increasingly focus on solvent-free reactive systems, lower-migration chemistries, and bio-attributed polyurethane formulations aimed at reducing environmental impact while maintaining performance [49]. Research also explores compatibilising strategies and debond-on-demand concepts to facilitate recycling without compromising laminate functionality [36]. These trends highlight how reactive adhesives, while essential for high-performance packaging, remain central to ongoing efforts toward more circular packaging materials.

#### 3.3.4. Water- and Solvent-Based Adhesives

Within the activation-mechanism perspective, water- and solvent-based adhesives form bonds primarily through the evaporation of a carrier phase that enables adhesive wetting, spreading, and interfacial contact prior to film formation. This activation route is widely used in packaging because it allows relatively simple processing conditions, good substrate wetting, and compatibility with established converting technologies [27].

Water-based systems are particularly prevalent in paper, paperboard, and fibre-based packaging, where substrate porosity facilitates rapid absorption and drying. Typical examples include starch- or dextrin-based adhesives, polyvinyl acetate dispersions, and other aqueous polymer systems used in labeling, corrugated packaging, and lamination of fibre-based structures [2,29]. Their solvent-free character from a regulatory standpoint and relatively low toxicity profiles makes them attractive for food-contact packaging, although moisture sensitivity and drying-time constraints can limit performance in high-barrier or humidity-variable environments.

Solvent-based adhesives, historically widespread in flexible packaging lamination, provide excellent wetting of low-energy polymer surfaces and enable uniform adhesive film formation even on non-porous substrates such as plastics, aluminum foils, or coated materials. However, environmental, safety, and regulatory concerns related to VOC emissions have progressively reduced their use, driving a transition toward water-based or solvent-free alternatives in many packaging sectors [4,8,28].

From a system perspective, dispersion-based adhesives offer advantages in processing simplicity and regulatory compliance but may introduce constraints related to drying energy, line speed, and sensitivity to ambient humidity. Their selection therefore reflects a balance between converting efficiency, substrate compatibility, functional performance requirements, and environmental considerations [27]. Ongoing developments increasingly target low-VOC formulations, improved water resistance, and hybrid systems combining aqueous processing with enhanced durability, in line with broader sustainability and circularity objectives in packaging design.

### 3.4. Functional Application Domains in Packaging

Although chemical origin and activation mechanisms represent primary classification axes for packaging adhesives, functional application rarely constitutes an independent dimension. In practice, application domains emerge from the combined effects of material chemistry, processing route, substrate compatibility, and performance requirements. Tie-layer use illustrates this coupling particularly well: although often discussed in terms of formulation, tie layers are primarily an application-driven interfacial solution enabling multilayer architectures by compatibilising otherwise incompatible materials [50,51].

Labeling adhesives are commonly associated with pressure-sensitive systems or water-based dispersions, reflecting the need for rapid application, controlled removability, and compatibility with recycling processes. Multilayer lamination, by contrast, frequently relies on reactive or hot-melt technologies capable of ensuring durable interfacial bonding under mechanical, thermal, and environmental stresses. Fibre-based packaging applications often favour bio-derived dispersions or starch-based adhesives due to their compatibility with paper recycling streams and aqueous processing routes [52].

These examples illustrate that functional application represents the practical manifestation of the multidimensional framework outlined in Section 2, rather than a standalone classification. Adhesive selection in packaging is therefore a coupled optimisation process, where chemistry, processing conditions, performance requirements, and end-of-life considerations converge within specific packaging applications.

### 3.5. Performance Metrics and System Constraints for Packaging Adhesives

While functional role and material–processing dimensions provide a structural framework for classifying adhesive technologies in packaging, practical material selection ultimately depends on performance metrics and system-level constraints associated with converting operations, service conditions, regulatory compliance, and end-of-life scenarios.

**Figure 10:**
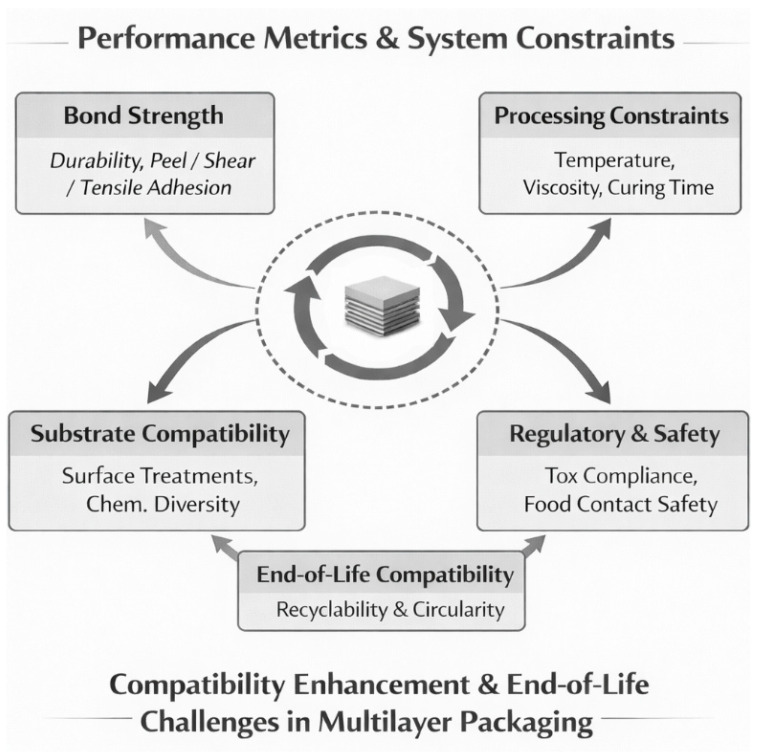
Performance metrics and system constraints governing adhesive selection in packaging applications. Adhesive performance in packaging systems results from the combined influence of bonding strength, processing constraints, substrate compatibility, regulatory and safety requirements, and end-of-life considerations. These interdependent factors determine material selection, processing feasibility, laminate durability, and recyclability in multilayer packaging structures.

These aspects are summarised schematically in Figure 10, which highlights the multidimensional performance metrics and system-level constraints governing adhesive selection in packaging applications.

A primary performance parameter is bonding strength, typically assessed through peel, shear, or tensile adhesion tests depending on the packaging configuration [31]. Adequate bond strength is required not only to ensure laminate integrity or label retention during distribution and handling, but also to maintain structural cohesion under thermal cycling, humidity exposure, mechanical loading, and long-term storage conditions. Durability and ageing resistance are therefore critical complementary metrics, particularly for multilayer flexible packaging and reusable packaging systems.

**Processing-related constraints** constitute a second major dimension. Adhesives must operate within defined process windows, including application temperature, viscosity range, open time, curing or setting time, and compatibility with industrial line speeds. These factors directly affect manufacturing efficiency, defect rates, and operational safety. For dispersion-based systems, drying kinetics and ambient humidity control become key considerations, whereas reactive adhesives require careful management of curing conditions and residual-reactive-species control, particularly in food-contact applications [32].

**Substrate compatibility** represents another essential criterion. Adhesives must ensure reliable wetting and adhesion across chemically diverse materials such as polyolefins, polyesters, metals, glass, coated papers, and composite barrier structures. Surface treatments, primers, and formulation adjustments are often required to achieve consistent adhesion, especially in multilayer packaging where mismatched surface energies and mechanical properties are common [33].

**Regulatory and safety constraints** increasingly influence adhesive selection, particularly in food, pharmaceutical, and cosmetic packaging. Migration limits, toxicological compliance, odour neutrality, and compatibility with recycling streams impose additional formulation requirements that may restrict otherwise high-performance adhesive chemistries [32,53,54].

Finally, **end-of-life compatibility** has emerged as a critical system constraint. Strong adhesive bonding, multilayer complexity, and chemical incompatibilities can hinder recycling processes, interfere with material separation, or contaminate recycling streams. This creates an inherent trade-off between packaging performance during use and circularity objectives [41]. Current research therefore focuses on compatibilising adhesive formulations, wash-off and removable systems, debond-on-demand interfaces, and bio-based or lower-impact chemistries aimed at reconciling functionality with recyclability and sustainability requirements [39].

This system-level perspective provides the basis for the discussion of sealing technologies in the following section, where similar multidimensional constraints govern the design of packaging closures and barrier continuity.

## 4. Sealant Systems for Packaging

Section 4 explicitly addresses the performance and constraint dimension of the pro-posed framework, analysing how material selection and functional roles translate into system-level requirements.

Sealants are interfacial materials that govern hermetic closure, barrier continuity at seams, and seal integrity under the thermal and mechanical loads associated with converting, filling, transport, and storage. In flexible and semi-rigid packaging formats, seam performance often constrains the reliability of the entire pack, since leakage resistance and shelf-life retention depend directly on seal formation and stability [3,4,20,34,55]. Recent reviews focused specifically on flexible packaging sealants further highlight the importance of seal material selection, sealing mechanisms, and process optimisation in determining packaging reliability and performance [21].

From an engineering standpoint, sealants cannot be assessed independently of substrate architecture and sealing conditions. Seal initiation temperature (SIT), hot-tack behaviour, seal strength, and sealing-window width are coupled to polymer chemistry, layer thickness, thermal history, and line settings (temperature–pressure–dwell time), so optimisation typically implies trade-offs between process robustness, barrier continuity at the seam, and end-of-life compatibility [2,7,21]. In addition, packaging represents a major share of the global adhesives and sealants market (≈43%), reflecting the broad diversity of sealant solutions tailored to specific converting lines and package formats [56].

**Figure 11:**
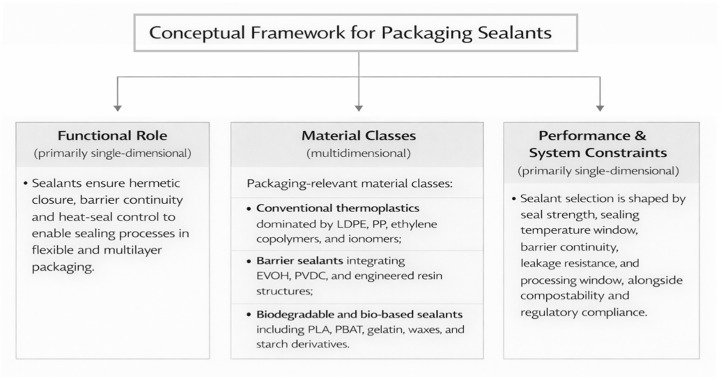
Conceptual framework for packaging sealants. Sealant selection is framed along three complementary axes: (i) functional role (primarily monodimensional), focused on hermetic closure, barrier continuity, and heat-seal control; (ii) material classes (multidimensional), grouping packaging-relevant sealants into conventional thermoplastics, multilayer barrier sealants, and biodegradable/bio-based systems; and (iii) performance and system constraints (primarily single-dimensional), driven by seal strength, sealing temperature window, barrier continuity, leakage resistance, and processing window.

These coupled relationships are synthesised in Figure 11, which frames packaging sealants along (i) functional role at the closure interface, (ii) material classes adopted in packaging, and (iii) performance and system constraints that govern industrial feasibility and package reliability. Building on this framework, the following subsections discuss conventional thermoplastic sealants, multilayer/barrier solutions, biodegradable and bio-based alternatives, and advanced functional sealing concepts, before addressing system-level implications for recyclability and circular design.

### 4.1. Functional Roles of Sealants in Packaging

Within packaging architectures, sealants perform a limited set of recurring functions that can be defined independently of the specific formulation. Their primary role is to enable hermetic closure and ensure barrier continuity at seams, i.e., the maintenance of gas and vapour protection across the package interface where the barrier is inherently interrupted by the sealing operation [3,4,20,55]. Reviews specifically focused on seal materials in flexible packaging further emphasise that seal integrity and seam continuity are often the primary determinants of package reliability and shelf-life stability [21].

A second functional dimension concerns heat-seal control under line conditions, where sealants must provide adequate seal strength while accommodating variability in temperature, pressure, and dwell time, and preserving seal integrity under handling and early post-seal loading through hot-tack performance [2,7,21]. Experimental and modelling studies also highlight the strong dependence of seal performance on material rheology, interfacial adhesion, and process conditions [34].

Finally, sealants increasingly act as system-level enablers or constraints for recyclability and circular design, since the sealing layer directly affects mono-material compatibility, separability, and material purity at end of life [57]. These functional roles provide the basis for the material- and mechanism-oriented discussion in the following section, which groups sealants into conventional thermoplastic families, barrier-oriented multilayer solutions, biodegradable/bio-based systems, and advanced functional sealing technologies.

### 4.2. Material Chemistry and Sealing Mechanisms

Following the functional perspective outlined above, sealant systems can be interpreted in terms of their material chemistry and sealing mechanisms, which govern seal formation, processing robustness, and integration within packaging architectures. In contrast to structural adhesives, whose primary function is load-bearing bonding between substrates, packaging sealants operate mainly through thermally activated interfacial fusion and viscoelastic flow, while selected technologies rely on pressure-activated or triggerable interfaces to enable rapid closure under industrial converting conditions [34].

From a materials standpoint, sealants span a broad spectrum ranging from conventional thermoplastic polyolefins widely used in flexible packaging, to engineered barrier-oriented multilayer solutions designed to preserve protection at seams, to biodegradable and bio-based formulations developed to address sustainability requirements, and finally to advanced functional sealing systems tailored for specific processing, performance, or circularity constraints. These categories are not strictly distinct material classes but application-driven technological solutions whose selection depends on sealing conditions, substrate compatibility, barrier requirements, and end-of-life considerations [58].

The following subsections therefore discuss the principal sealant families employed in packaging, focusing on sealing mechanisms, processing implications, and architectural integration.

This complexity can also be interpreted in light of broader adhesive–sealant taxonomies. For instance, Ref. [19] organizes adhesive and sealant systems according to chemical composition, application function, and activation/curing mechanism, distinguishing thermoplastic versus thermoset families, reactive chemistries, and physically bonded systems. This perspective is useful in packaging, where sealing performance remains tightly coupled to process conditions and substrate architectures.

**Figure 12:**
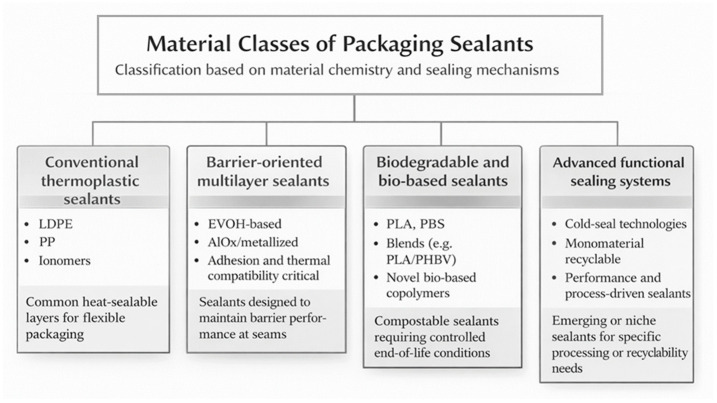
Material classes of packaging sealants (packaging-oriented classification). Overview of major sealant families discussed in Section 4.2, including conventional thermoplastic sealants, barrier-oriented multilayer solutions, biodegradable/bio-based sealants, and advanced functional sealing systems, highlighting their typical role in packaging architectures and the main system constraints affecting their selection.

In the present review, this general viewpoint is translated into a packaging-oriented classification that groups sealants into conventional thermoplastic families, barrier-oriented multilayer solutions, biodegradable/bio-based systems, and advanced functional sealing technologies. These relationships are summarised schematically in Figure 12, which positions the main sealant families discussed in Section 4.2 within the packaging context and anticipates the corresponding subsections.

#### 4.2.1. Conventional Thermoplastic Sealants

Within the material-oriented framework introduced above, conventional thermoplastic sealants represent the dominant class of sealing materials in packaging, particularly in flexible and semi-rigid formats. Polyolefin-based materials, including LDPE, LLDPE, metallocene-catalysed polyethylene, polypropylene, and ethylene copolymers, are widely adopted due to their broad sealing windows, good hot-tack performance, and compatibility with high-speed heat-sealing operations [2,6,59]. Recent reviews specifically focused on seal materials in flexible packaging confirm the continuing industrial predominance of polyolefin-based sealants due to their favourable balance between processability, sealing reliability, and cost efficiency [21].

Their sealing performance derives from thermally activated viscoelastic flow and interfacial fusion under heat and pressure, forming cohesive bonds upon cooling [34]. Key performance parameters include SIT, maximum seal strength, and tolerance to variability in temperature, pressure, and dwell time. In industrial practice, materials with wider sealing windows are preferred because they reduce sensitivity to process fluctuations and equipment heterogeneity [4].

Despite favorable processing characteristics, conventional thermoplastic sealants often exhibit limited intrinsic gas and vapour barrier performance. Materials such as low-density polyolefins typically provide poor barrier properties and therefore require multilayer packaging structures in which dedicated barrier layers compensate for permeability [58,60]. Although derived from petrochemical sources and generally non-compostable, these thermoplastics maintain industrial dominance. Their seamless integration into existing production lines, high throughput, and reliable performance under diverse environmental conditions continue to outweigh sustainability concerns in many applications [57].

**Figure 13:**
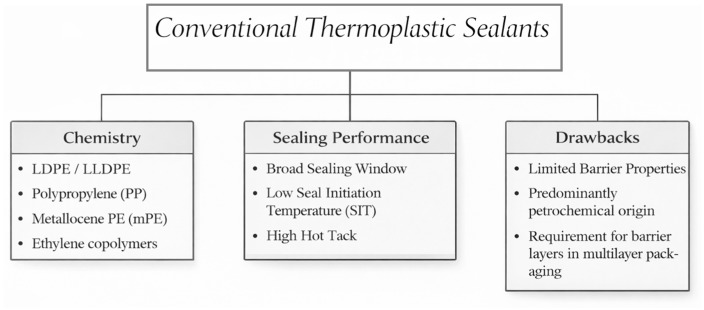
Conventional thermoplastic sealants: material families, sealing performance, and main limitations. Schematic overview of polyolefin-based thermoplastic sealants commonly used in packaging, highlighting representative material chemistries, typical sealing performance characteristics (sealing window, SIT, hot tack), and key limitations related to barrier properties and sustainability considerations.

The principal characteristics of conventional thermoplastic sealants, including their chemical variants, sealing performance attributes, and inherent limitations, are summarised in Figure 13.

#### 4.2.2. Multilayer and Barrier Sealants

From a material and architectural perspective, sealants are frequently integrated into multilayer packaging structures to meet demanding shelf-life and protection requirements. In these systems, the sealant layer must not only provide reliable closure but also maintain barrier continuity at the seam, preventing preferential permeation paths that could compromise package integrity [7,21,55].

Barrier-oriented sealant systems may involve modified polyolefins, copolymer blends, or engineered multilayer designs incorporating high-barrier polymers (e.g., EVOH or PVDC) as dedicated barrier layers combined with sealant layers [61]. While these approaches significantly improve gas and vapour resistance, they introduce additional constraints related to sealability, thermal sensitivity, and compatibility with recycling processes [58]. A central design challenge in high-performance packaging therefore lies in balancing barrier enhancement with sealing robustness.

During multilayer design and lamination, sealing temperature compatibility between layers, recyclability issues associated with material heterogeneity, and compliance with food contact regulations must all be considered [3,59]. Despite recyclability concerns, these multilayer sealant systems remain essential for premium packaging formats such as retort pouches, modified atmosphere packaging (MAP), and multilayer thermoformed trays. They enable extended shelf life and performance levels generally unattainable with single-material solutions [62].

**Figure 14:**
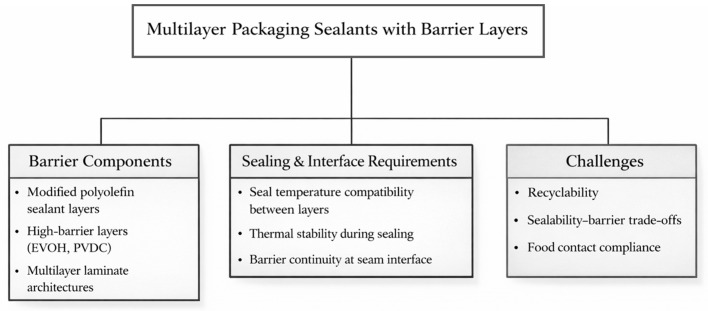
Multilayer and barrier sealants in packaging applications. Conceptual overview of multilayer barrier sealants, highlighting barrier enhancement strategies, sealing requirements at the package seam, and key challenges related to recyclability, sealability trade-offs, and food contact compliance.

Figure 14 summarises the principal barrier components, sealing and interface requirements, and system-level challenges associated with multilayer packaging sealants incorporating dedicated barrier layers.

#### 4.2.3. Biodegradable and Bio-Based Sealants

The transition toward compostable and bio-based packaging has stimulated growing interest in biodegradable sealant systems derived from biopolymers such as PLA, PBS, PBAT, and protein- or polysaccharide-based formulations. These materials are primarily adopted in applications where alignment with compostability standards or renewable sourcing is prioritised, and are specifically intended for use with bio-based and compostable packaging substrates—including PLA-based films, cellulose-based structures, and starch-composite materials—where system-level end-of-life compatibility requires that both substrate and sealing layer be processable within the same composting or biodegradation pathway [7,8,30,58,63,64,65]. They are essential for the development of certified compostable packaging formats under EN 13432 [66] or ASTM D6400 [67] standards and are increasingly found in films labelled as “home-compostable” or suitable for organic waste streams.

Due to their polymer chemistry and thermal behaviour, biodegradable sealants often exhibit narrower sealing windows, reduced hot-tack strength, and greater sensitivity to moisture and temperature compared to conventional polyolefin-based systems [63]. These characteristics impose constraints on line speed, process stability, and application scope, particularly in high-throughput industrial environments [34]. As a result, biodegradable sealants are frequently used in niche applications or in combination with adapted processing conditions to ensure acceptable performance.

**Figure 15:**
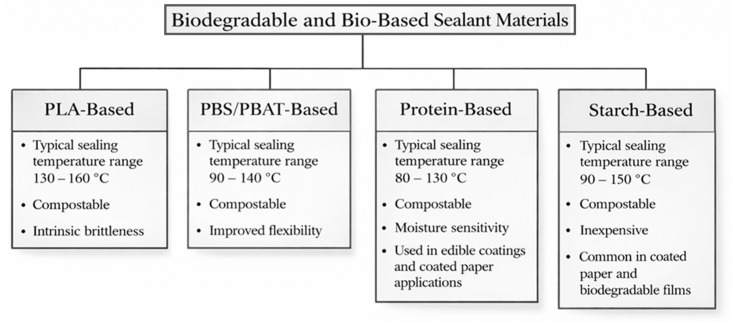
Biodegradable and bio-based sealant materials for packaging applications. Schematic classification of biodegradable and bio-based sealant families, including PLA-based systems, PBS/PBAT-based blends, protein-based sealants, and starch-based sealants. The figure highlights typical sealing temperature ranges, compostability attributes, and key material limitations, summarising the main performance trade-offs and end-of-life implications relevant to packaging integration.

The integration of biodegradable sealants within multilayer or hybrid packaging structures further complicates end-of-life scenarios, as mismatches in degradation behaviour or recycling compatibility may undermine sustainability objectives [55,58]. Figure 15 summarizes the main classes of biodegradable and bio-based sealing materials: PLA, PBS/PBAT, protein- and starch-based, and their main characteristics.

#### 4.2.4. Advanced and Functional Sealing Systems

Beyond conventional heat-sealing, advanced sealing concepts have been developed to address specific processing constraints, energy demands, or circularity targets. Examples include cold-seal systems, induction sealing, ultrasonic sealing, and triggerable or reversible interfaces designed to enable controlled opening or selective delamination [21,68,69].

These approaches may reduce thermal load on sensitive products, improve energy efficiency, or facilitate layer separation at end of life. However, they typically require dedicated equipment, tighter process control, or tailored material formulations, which can limit adoption outside targeted applications. Advanced sealing systems are therefore evaluated not only in terms of seal integrity but also by integration cost and compatibility with existing converting lines [1,2,34].

Recent work also includes material-level and process-level innovations that improve sealability and robustness in bio-based laminates. For instance, [70] reported PLA/PBS-based films reinforced with modified zeolite nanoparticles, improving moisture barrier and sealing performance in compostable structures. In parallel, Ref. [71] investigated the heat-sealing behaviour of bio-based nanocomposite films across a range of temperatures, revealing how varying the sealing temperature affects their sealing properties for food packaging applications. This supports a more reliable translation from laboratory development to industrial implementation. Overall, advanced sealing technologies tend to be deployed as application-specific solutions where their benefits justify additional complexity [55].

**Figure 16:**
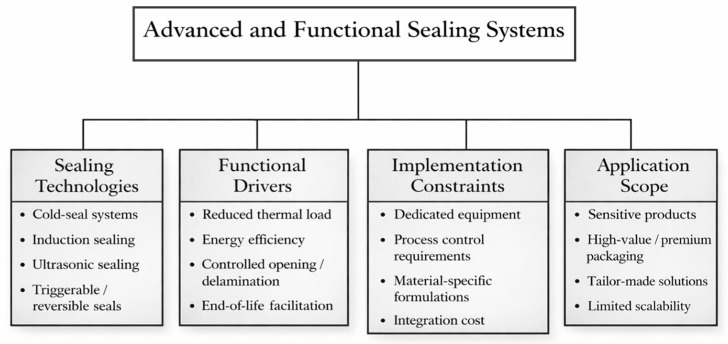
Advanced and functional sealing systems in packaging applications. Schematic overview of advanced sealing approaches beyond conventional heat sealing. The figure summarizes major sealing technologies, their primary functional drivers, implementation constraints, and typical application scope, highlighting their role as targeted, application-specific solutions rather than universal replacements for conventional sealants.

Figure 16 shows a structured representation of advanced and functional sealing systems, organized into four macro-areas: sealing technologies, performance factors, implementation constraints and field of application.

Despite their functional appeal, advanced sealing technologies face significant barriers to mainstream adoption. Ultrasonic and laser sealing require capital-intensive equipment and process re-engineering that limit their integration into existing converting lines [59]. Induction sealing is restricted to metal-containing substrates, while cold sealing formulations raise unresolved migration safety concerns in food-contact applications [72]. Moreover, the available literature on long-term performance and industrial-scale reliability remains limited, and systematic comparative data under standardised packaging conditions are still largely absent.

### 4.3. Performance Metrics and System Constraints for Packaging Sealants

Beyond material chemistry and sealing mechanisms, the effectiveness of packaging sealants must be evaluated through performance metrics and system-level constraints that ultimately determine their suitability in real packaging applications. Seal integrity is commonly assessed through parameters such as seal strength, hot-tack resistance, and SIT, which collectively define the operational sealing window and process robustness. Experimental studies have shown that sealing performance is highly sensitive to processing conditions, material formulation, and contamination at the sealing interface, all of which may significantly affect leak tightness and mechanical reliability [73].

Barrier performance at the seal interface represents another critical aspect. Even when high-barrier materials are incorporated in multilayer packaging, seal regions may become preferential permeation pathways if sealing parameters are suboptimal or material compatibility is insufficient [74].

System-level constraints further complicate sealant selection. Industrial packaging lines impose limitations related to sealing speed, thermal uniformity, dwell time variability, and compatibility with existing converting equipment. Heat-sealing behaviour is strongly influenced by rheological properties, thermal response, and interfacial adhesion phenomena, making the sealing process intrinsically sensitive to both material design and operational conditions [34]. These constraints often require compromises between sealing robustness, process efficiency, and material sustainability.

In multilayer packaging architectures, interactions between sealants, adhesives, and barrier layers must also be considered to ensure consistent sealing behaviour and long-term stability. From a circularity perspective, multilayer configurations can introduce additional challenges for recycling and material recovery, particularly when incompatible polymers are combined or when sealant layers hinder separation processes [58].

**Figure 17:**
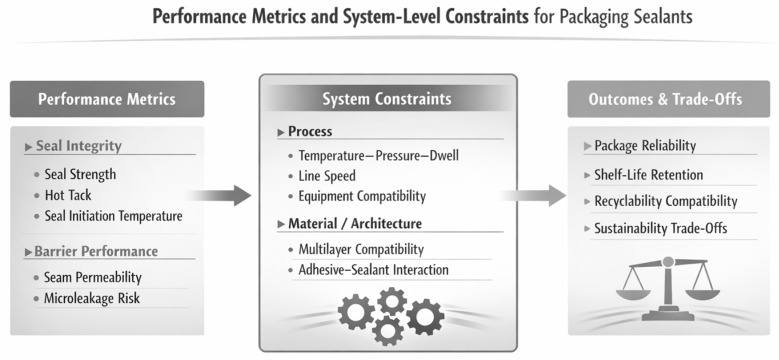
Performance metrics and system-level constraints governing packaging sealant selection. Conceptual framework illustrating the interdependence between sealing performance metrics (seal strength, hot tack, sealing temperature window), processing constraints (line conditions, thermal control, equipment compatibility), packaging architecture factors (multilayer integration, adhesive–sealant interactions), and circularity considerations.

Regulatory requirements, especially in food-contact packaging, introduce further constraints related to migration limits, chemical compatibility, and thermal stability during processing [54]. Consequently, sealant design increasingly involves balancing sealing performance, processability, barrier requirements, and end-of-life considerations, highlighting the inherently trade-off-driven nature of modern packaging sealant systems (Figure 17).

### 4.4. System-Level Constraints and End-of-Life Implications

Sealant selection in packaging cannot be considered solely in terms of sealing performance or material chemistry, as sealing layers strongly influence the end-of-life behaviour of packaging systems. Even when present in relatively small quantities, sealants may affect recyclability, compostability, and material separation efficiency, particularly in multilayer structures where chemical incompatibility can lead to contamination of recycling streams [58,75].

From a circularity perspective, increasing attention is therefore being paid to mono-material sealing solutions, compatibilised sealant formulations, and selectively removable sealing interfaces designed to facilitate material recovery [76]. For example, sealants based on compatible polyolefin chemistries are increasingly used in polyethylene-dominated flexible packaging to maintain recyclability without compromising sealing performance [59]. Similarly, reversible or triggerable sealing interfaces and delamination strategies have been explored to enable layer separation in complex laminates, supporting more efficient material recovery and circular packaging design [39].

However, trade-offs remain unavoidable. Sealants optimised for high barrier performance or extended shelf life may introduce material heterogeneity that complicates recycling, whereas fully recyclable or compostable sealants may impose constraints on processing robustness, sealing strength, or environmental resistance [21,58,73]. These competing requirements highlight the importance of integrated packaging design, where sealants, adhesives, substrates, and barrier layers are selected jointly rather than independently.

**Figure 18:**
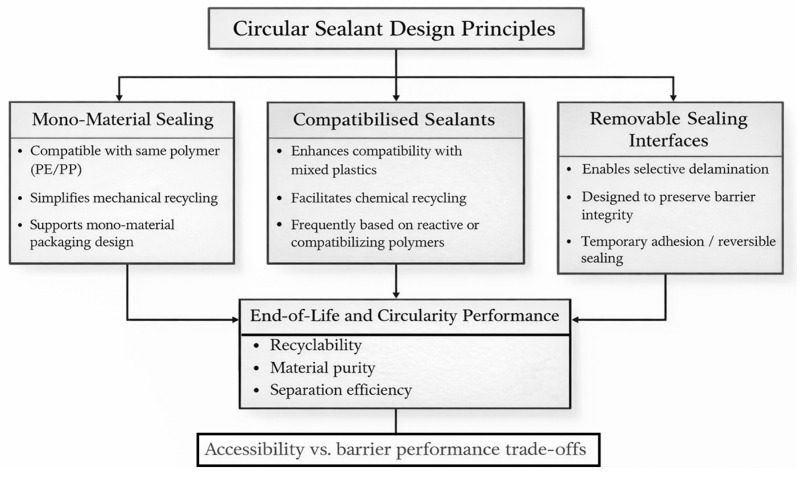
System-level sealant design strategies and end-of-life implications in packaging. Conceptual framework linking circular sealant design principles to three key strategies—mono-material sealing, compatibilised sealants, and removable sealing interfaces—and to their effects on end-of-life and circularity performance (recyclability, material purity, and separation efficiency).

The system-level implications of sealant selection are summarised in Figure 18, which links sealing technologies to circular design strategies, recyclability pathways, and end-of-life performance considerations.

These considerations reinforce the need to interpret adhesive and sealant systems through the combined lens of functional role, material/activation mechanisms, and performance constraints, as proposed in the three-dimensional framework.

## 5. Comparative Performance Metrics and System-Level Trade-Offs

Unlike bulk materials, which can often be compared using intrinsic properties (e.g., mechanical strength or thermal stability), adhesives and sealants must be assessed using application-specific indicators that describe interface formation and integrity within a given packaging architecture. Key metrics therefore include bond strength and seal strength, SIT, hot-tack behaviour, and the width of the processing window [21,34]. These indicators are not universal constants: they vary with substrates, layer sequence, and line settings.

Accordingly, this section provides a comparative framework based on the performance indicators most consistently reported in packaging-oriented studies, industry practice, and technical references. The aim is to support cross-family comparison through a semi-quantitative reading of the literature, highlighting typical performance ranges and the main trade-offs that guide material selection in real packaging systems.

### 5.1. Comparative Metrics for Adhesives

In packaging applications, adhesives are generally evaluated through functional performance indicators that reflect their behaviour within packaging systems rather than intrinsic material properties alone. Because adhesive performance depends strongly on substrates, converting conditions, service environment, and end-of-life requirements, comparisons across adhesive types are typically based on application-driven metrics describing interface reliability, processing compatibility, and sustainability implications [6,77].

Moreover, commonly used adhesive categories do not always correspond to strictly homogeneous chemical families. Some classifications refer to material origin (e.g., natural vs. synthetic adhesives), others to activation or processing mechanisms (e.g., hot-melt or pressure-sensitive systems), and others to functional roles within packaging architectures. Consequently, meaningful comparison is better achieved through functional performance indicators rather than purely chemical classification [77].

The comparative analysis presented here therefore refers exclusively to the adhesive categories introduced in Section 3. The objective is not to provide an exhaustive taxonomy of adhesive technologies, but to compare how representative packaging interface solutions perform from a functional, system-level perspective relevant to packaging conversion, use, and disposal.

Within packaging-oriented studies and industrial practice, five metrics are commonly used to characterise adhesive performance:

- Functional bond reliability.

This describes the ability of the bonded interface to maintain integrity under mechanical loads, temperature fluctuations, humidity exposure, and handling stresses typical of packaging service conditions. The indicator reflects practical interface durability rather than intrinsic adhesive strength alone, since performance depends strongly on substrate combinations and joint geometry [6,24].

- Processing compatibility.

This refers to how effectively an adhesive integrates into converting operations, including application conditions, curing or setting behaviour, robustness at industrial line speeds, and operational stability. In packaging manufacturing, process reliability is often as critical as ultimate bond strength [6].

- Substrate versatility.

This indicates the capability to bond a wide range of packaging materials, including polymers, paper and board, metals, coated films, and multilayer structures. Surface wetting behaviour and compatibility with treated or functionalised substrates are central aspects of this metric [77].

- Service resistance.

This metric describes the stability of the bonded interface during storage, transport, and use, considering temperature variations, moisture exposure, mechanical stresses, and interactions with packaged contents. Regulatory compliance, including food-contact considerations when applicable, is often associated with this performance aspect [6].

- End-of-life compatibility.

This refers to the influence of adhesives on recycling, compostability, delamination, wash-off removal, or material separation. Because packaging sustainability increasingly depends on circular design strategies, this metric has become a key consideration in adhesive selection and formulation [39].

These indicators provide a functional framework for comparing the adhesive solutions discussed in Section 3. The qualitative comparison summarised in Table 1 is organised according to the same classification axes previously introduced (functional role, chemical origin, activation or processing route, and application context), ensuring methodological consistency across the review.

The qualitative comparison reported in Table 1 is based on a synthesis of information extracted from packaging-oriented handbooks, technical classification manuals, and review literature dealing with adhesive performance in packaging systems (e.g., [2,6,22,29,77]). The reported levels should therefore be interpreted as comparative functional trends rather than absolute material properties.

### 5.2. Interpretation of Comparative Trends for Adhesives

The qualitative comparison reported in Table 1 confirms that adhesive performance in packaging is largely governed by system-level constraints rather than intrinsic material properties alone. Differences among adhesive classes primarily reflect trade-offs between bond reliability, processing requirements, substrate compatibility, environmental resistance, and end-of-life considerations.

Synthetic and reactive adhesive systems generally provide higher bond integrity and environmental durability, particularly in demanding multilayer or flexible packaging structures. However, these systems may present limitations in terms of recyclability or material separation, especially when strong interfacial bonding complicates delamination processes [8,21].

Conversely, natural and bio-based adhesives tend to show lower mechanical robustness and environmental resistance, but they often offer advantages in terms of compatibility with paper recycling streams, compostable packaging formats, or circular design strategies [29,37]. Their adoption is therefore frequently driven by sustainability requirements rather than maximum performance.

Process-driven adhesive technologies such as hot-melt, pressure-sensitive, or water-/solvent-based systems are typically selected primarily for converting efficiency, operational reliability, and substrate-specific compatibility. In these cases, processing constraints may outweigh purely mechanical considerations in adhesive selection [25].

Finally, functional interlayers such as tie layers or removable/debond-on-demand systems highlight the increasing importance of interface engineering in packaging design. These solutions are often tailored to specific multilayer architectures or circularity requirements, reinforcing the idea that adhesive performance must be assessed within the broader packaging system rather than at the material level alone [78].

### 5.3. Comparative Metrics for Sealants

Packaging sealants are evaluated through performance indicators directly linked to seam formation and integrity, which frequently represent the limiting factor for package reliability and barrier preservation. Unlike bulk film properties, seal performance depends strongly on sealing conditions, including temperature, pressure, dwell time, and material architecture. Consequently, sealant performance is typically assessed through functional metrics reflecting processability and in-service reliability rather than intrinsic material properties alone [21,34].

The most commonly used metrics include:

- Seal initiation temperature (SIT).

The minimum temperature at which a measurable seal can be formed under defined process conditions. SIT is widely used as a practical indicator of process efficiency and sealing robustness [34].

- Seal strength.

The maximum force sustained by the sealed joint before failure. Values depend strongly on test configuration, sealing conditions, and substrate combination [21].

- Hot-tack behaviour.

Resistance of the seal immediately after formation, before complete cooling. This parameter is particularly relevant for vertical form–fill–seal operations and high-speed converting [79].

- Sealing window width.

The temperature interval over which acceptable sealing performance is achieved, reflecting tolerance to process variability [34].

- Barrier continuity at the seam.

Ability of the seal region to maintain gas and vapour barrier performance without preferential leakage paths, often a critical aspect in multilayer packaging [21].

- Resistance to processing and use conditions.

Seal stability under pasteurisation, retort sterilisation, aggressive contents, and mechanical handling [34].

- End-of-life compatibility.

Influence of sealant selection on recyclability, compostability, and material separation in circular packaging systems [21].

The qualitative comparison reported in Table 2 is derived from a synthesis of packaging-focused reviews and experimental studies dealing with seal performance, heat-sealing behaviour, and seam integrity, particularly those addressing flexible food packaging systems [21,34,58,79].

### 5.4. Interpretation of Comparative Trends for Sealants

Although numerical values vary significantly with sealing conditions, some consistent trends emerge from the literature.

Polyolefin-based sealants typically show relatively low SIT values, often in the range of roughly 90–130 °C, combined with seal strengths frequently between 2 and 6 N/15 mm under standard laboratory conditions. Their wide sealing window and good hot-tack behaviour explain their extensive use in high-speed packaging operations [21,34].

Barrier-oriented multilayer systems, including structures incorporating EVOH or metallised layers, generally require higher sealing temperatures, typically around 110–150 °C, and may exhibit narrower process windows due to the need to preserve barrier layers during sealing. However, they provide improved seam barrier continuity compared with simpler polyolefin systems [21].

Biodegradable sealants such as PLA-, PBS-, or PBAT-based systems tend to require higher sealing temperatures, often exceeding 120–170 °C, and may show lower hot-tack and seal strength values, typically around 1–4 N/15 mm. These limitations are balanced by improved end-of-life compatibility in compostable packaging applications [34].

Finally, advanced sealing concepts—including cold-seal adhesives, induction sealing, ultrasonic sealing, and reversible or triggerable systems—exhibit highly application-dependent performance. Their advantages are generally related to specific process or sustainability requirements rather than universal improvements in mechanical performance.

Building on the separate comparative analyses of adhesive systems (Table 1) and sealant systems (Table 2), Table 3 provides an integrated system-level synthesis of the main trade-offs emerging across both interfacial material classes. Rather than introducing a direct numerical benchmark, the table summarises the relative balance between interfacial performance, process robustness, and circularity compatibility, consistently with the framework-oriented scope of Part I and with the methodological variability discussed in Section 1.2.

### 5.5. Adhesives–Sealants Interplay in Multilayer Systems

In multilayer packaging structures, adhesives and sealants do not operate independently but jointly determine mechanical integrity, barrier continuity, and failure behaviour under service conditions. As widely recognised in packaging technology studies, overall package performance is often controlled by interfacial behaviour rather than solely by the intrinsic properties of individual layers [6,21,80].

For example, even highly efficient barrier layers may lose effectiveness if seal continuity is compromised at the package seam, where preferential permeation paths can develop [34]. Conversely, very strong adhesive bonding can hinder material separation during recycling, creating a trade-off between mechanical performance and circularity that is increasingly discussed in the context of sustainable packaging design.

These interactions highlight the need for system-level optimisation in packaging design, where adhesive and sealant selection is coordinated with substrate architecture, converting processes, and end-of-life strategy. Hybrid interfacial solutions positioned between bonding and sealing functions—such as heat-sealable adhesives or compatibilising tie layers—are increasingly explored to simplify multilayer structures and improve recyclability while maintaining functional performance [6,21,58].

### 5.6. Design Trade-Offs and Selection Criteria

The comparative framework presented in this section indicates that no single adhesive or sealant system simultaneously maximises functional performance, process robustness, and sustainability. Material selection in packaging therefore inevitably involves balancing competing requirements that arise from processing conditions, service performance, regulatory constraints, and end-of-life considerations [2,6,21].

In practical packaging applications, trade-offs commonly emerge between process efficiency and thermal or environmental stability. Adhesives or sealants optimised for high-speed converting operations, for example, may exhibit reduced resistance to elevated temperatures, aggressive contents, or long-term environmental exposure [2]. Similarly, strong bonding or sealing performance often conflicts with recyclability, since highly durable interfacial layers can hinder material separation or contaminate recycling streams [21].

Barrier performance represents another critical dimension. Multilayer architectures designed to maximise gas or moisture protection frequently rely on specialised adhesives and sealants that introduce material heterogeneity, potentially complicating recycling or compostability pathways [8]. Conversely, simplified mono-material solutions that facilitate circularity may impose limitations on barrier performance, processing robustness, or long-term durability [57].

These trade-offs underscore the inherently systemic nature of packaging design, requiring adhesives and sealants to be selected in tandem with substrate choice, converting processes, regulatory demands, and planned end-of-life pathways. Explicit recognition of these interdependencies not only supports more informed material selection but also identifies key areas—from debond-on-demand interfaces to compatibilised multilayer systems and bio-based materials—where future innovation can help align performance with circularity goals [6,21].

Overall, the framework proposed here provides a structured basis for interpreting adhesive and sealant selection in packaging while remaining adaptable to future quantitative refinement as more standardised and application-specific data become available.

## 6. Regulatory, Safety and Circularity Aspects

While food-contact applications generally represent the most stringent regulatory context—owing to the potential for chemical migration into consumable products—adhesives and sealants used in packaging are subject to a broader set of regulatory drivers extending beyond food packaging [32]. Environmental directives, occupational safety requirements, emissions regulations, and sector-specific standards (including pharmaceutical, cosmetic, and industrial packaging applications) all contribute to shaping formulation strategies, processing constraints, and end-of-life considerations [81]. Consequently, regulatory aspects of packaging adhesives cannot be interpreted solely within the food-contact framework but must be addressed within a wider cross-sectoral context.

**Figure 19:**
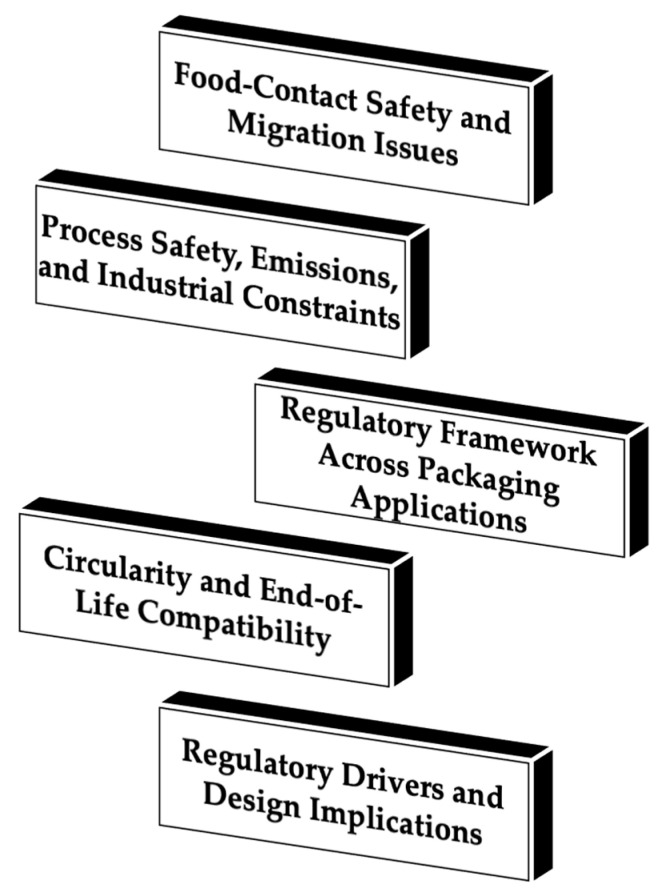
System-level roadmap of regulatory, safety, and circularity aspects for packaging adhesives and sealants. This diagram illustrates the integrated framework adopted in Section 6, highlighting the progression from food-contact safety and migration considerations (Section 6.1) to process-related safety and emissions constraints (Section 6.2), followed by the broader regulatory landscape across packaging applications (Section 6.3). Circularity and end-of-life compatibility aspects, including recyclability and compostability requirements (Section 6.4), are then considered as key design constraints. The final stage emphasises how evolving regulatory pressures and sustainability targets increasingly act as drivers for adhesive and sealant formulation, selection, and innovation (Section 6.5).

Accordingly, this section adopts a system-level perspective in which regulatory constraints are treated as design variables interacting with material chemistry, converting processes, and end-of-life scenarios. The discussion is organised as follows (Figure 19):

- Section 6.1 addresses food-contact compliance and migration-related safety aspects;
- Section 6.2 focuses on process-related safety constraints and emissions;
- Section 6.3 examines regulatory frameworks across packaging sectors;
- Section 6.4 discusses circularity aspects, including recyclability and compostability;
- Section 6.5 considers regulatory drivers, current gaps, and emerging challenges shaping innovation in adhesive and sealant technologies for packaging.

### 6.1. Food-Contact Compliance and Migration Issues

In packaging applications involving food, pharmaceuticals, or sensitive consumer products, adhesives and sealants are generally classified as indirect food-contact materials. Despite representing a relatively small fraction of total packaging mass, they can significantly influence product safety through the potential migration of chemical constituents across packaging layers. Residual monomers, additives, reaction by-products, degradation products, and non-intentionally added substances (NIAS) may migrate under specific conditions, particularly in multilayer systems where adhesives are positioned close to the product interface [82,83].

Migration is governed by diffusion, sorption, and permeation processes influenced by multiple factors, including adhesive chemistry, molecular weight distribution, polymer morphology, processing history, and the physicochemical characteristics of adjacent substrates and packaged products. Storage temperature, contact time, food composition (especially fat content), and surface-to-volume ratio also play a decisive role in determining migration rates and risk profiles [32]. These interactions highlight the intrinsically system-level nature of migration phenomena, which cannot be reliably assessed through material formulation alone.

Multilayer packaging structures introduce additional complexity because adhesives often function as lamination interfaces or tie layers. Recent analytical investigations using advanced chromatographic techniques have identified numerous migrating compounds in commercial multilayer packaging systems, including additives, degradation products, and solvent residues. Depending on exposure conditions, some substances may raise toxicological or sensory concerns, reinforcing the need for comprehensive analytical screening and risk-assessment procedures [84].

The increasing adoption of bio-based or biodegradable polymers introduces further safety considerations. Although often perceived as inherently sustainable, these materials may contain oligomers, additives, or degradation products capable of migration. Dedicated analytical approaches based on chromatographic and mass spectrometric techniques are therefore required to ensure consumer safety [85]. Bio-based origin does not automatically imply reduced migration risk.

From a toxicological perspective, migrants originating from adhesives, inks, coatings, or adjacent packaging layers may affect both food quality and consumer health. Plasticisers, stabilisers, residual solvents, and reaction by-products may cause sensory alterations, reduce shelf life, or contribute to cumulative exposure risks [72]. Regulatory compliance therefore requires integrated evaluation combining formulation control, migration testing under realistic conditions, analytical identification of IAS/NIAS, and risk assessment aligned with applicable legislation [86].

### 6.2. Process Safety, Emissions, and Industrial Constraints

Beyond food-contact safety, adhesives and sealants must comply with process-related safety requirements associated with industrial converting operations. These include volatile emissions during application and curing, worker exposure to chemical substances, thermal stability during sealing processes, and compatibility with high-throughput manufacturing conditions. Consequently, process safety represents a critical dimension of adhesive selection alongside chemical safety and regulatory compliance.

VOC emissions remain a major concern, particularly for solvent-based lamination adhesives widely used in flexible packaging [87]. Residual solvents may evaporate during coating, lamination, or curing operations, posing occupational exposure risks and environmental emissions while also contributing indirectly to contamination pathways within packaging structures [32]. Regulatory pressures have therefore encouraged a progressive transition toward solvent-free, water-based, and hot-melt adhesive systems.

Water-based adhesives significantly reduce VOC emissions but still require careful formulation control because surfactants, preservatives, and low-molecular-weight additives may influence environmental release, migration behaviour, or process stability. Hot-melt adhesives provide solvent-free processing advantages but introduce constraints related to temperature control, viscosity, substrate wettability, and thermal stability during sealing operations.

Polyurethane adhesives remain dominant in flexible multilayer packaging due to strong adhesion performance and chemical resistance. Solvent-free reactive systems, however, require careful control of curing kinetics and moisture sensitivity to avoid incomplete reactions or residual reactive species [88]. Processing conditions must therefore be optimised to ensure adhesion performance while minimising contamination risks.

Industrial safety considerations also include interactions between adhesive layers and adjacent packaging components. Substances originating from inks, recycled substrates, coatings, or polymer additives may diffuse into adhesive layers during processing. The increasing use of recycled materials amplifies variability in potential contaminants, reinforcing the need for comprehensive process monitoring across the packaging supply chain [72,89].

Overall, adhesive selection cannot be decoupled from manufacturing conditions. Emission control, occupational safety, curing behaviour, thermal stability, and compatibility with industrial converting technologies must be considered simultaneously within integrated packaging design strategies.

### 6.3. Regulatory Framework Across Packaging Applications

The regulatory framework governing adhesives and sealants used in packaging reflects the wide diversity of packaging applications, materials, and service conditions. Consequently, they are rarely addressed through dedicated legislation and are instead regulated indirectly through broader material regulations, chemical safety frameworks, sector-specific standards, and environmental directives. This regulatory positioning often results in partial harmonisation and heterogeneous compliance approaches across different packaging sectors [90].

In food-contact applications, adhesives typically fall within overarching food contact material (FCM) frameworks rather than adhesive-specific positive-list legislation. In the European Union, Regulation (EC) No. 1935/2004 establishes general safety requirements requiring that materials must not transfer constituents to food in quantities that could endanger human health or alter food composition or sensory properties. Complementary Good Manufacturing Practice provisions under Regulation (EC) No. 2023/2006 emphasise traceability, documentation, and controlled production conditions across the packaging supply chain. However, adhesives remain largely non-harmonised at EU level, with national regulations, industry guidelines, and risk-assessment methodologies often compensating for the absence of specific harmonised standards [90].

Recent European regulatory developments have further expanded the relevance of sustainability considerations in packaging design. The forthcoming PPWR introduces life-cycle-oriented requirements aimed at reducing packaging waste, improving recyclability, and supporting circular economy objectives [30]. Although not adhesive-specific, such policies directly affect adhesive and sealant selection because interfacial materials can influence material separation efficiency, recycling compatibility, and overall environmental performance of multilayer packaging systems.

Parallel regulatory initiatives addressing specific chemical classes also impact adhesive technologies. Increasing restrictions on substances such as bisphenols, fluorinated compounds (including PFAS), and other potentially hazardous additives reflect growing regulatory scrutiny of packaging-related chemical exposure [91,92]. These developments may affect adhesive formulations, coatings, and sealing materials, requiring continuous adaptation of material chemistry and compliance strategies.

The emergence of bio-based and sustainable adhesive formulations introduces additional regulatory complexity. While these materials are often promoted as environmentally favourable alternatives, their classification, safety assessment, and migration behaviour may remain insufficiently standardised. Recent analyses of sustainable packaging materials highlight the need for improved analytical screening methods, transparent chemical disclosure, and harmonised evaluation procedures to ensure both safety and regulatory compliance [93].

Taken together, these regulatory developments increasingly function as drivers of material innovation rather than mere compliance requirements, directly shaping adhesive formulation strategies and packaging design approaches.

### 6.4. Circularity and End-of-Life Compatibility

Recent investigations confirm that multilayer packaging remains a major technical obstacle to high-quality recycling and closed-loop material recovery [57,94].

Technological strategies aimed at improving recyclability include controlled interfacial adhesion concepts, selective adhesive placement, and water-soluble adhesive systems designed to facilitate delamination while maintaining packaging performance during service life. Experimental studies indicate that controlled reduction in interfacial adhesion can enable efficient material separation without compromising functionality [95].

Advanced recycling technologies are also emerging to address adhesive-related barriers. Chemical delamination processes based on selective solvent systems or targeted depolymerisation reactions have demonstrated potential for recovering intact polymer layers from multilayer packaging without significant degradation. Selective aminolysis of polyurethane and acrylate adhesive layers, for example, enables simultaneous delamination and deinking while preserving polymer quality [96].

Another promising development involves stimuli-responsive or debondable adhesives engineered for circular packaging systems. Light-responsive thermoplastic adhesives incorporating photolabile groups have demonstrated controlled delamination upon irradiation while maintaining adequate adhesion during product use. Preliminary environmental assessments suggest potential life-cycle benefits after limited reuse cycles [97].

Life-cycle assessment (LCA) evidence on bio-based adhesives and sealants relative to their synthetic counterparts reveals a more nuanced picture than sustainability narratives often suggest. Comparative LCA studies indicate that bio-based adhesive systems can achieve meaningful reductions in greenhouse gas emissions relative to petrochemical equivalents, with reported reductions in the range of 19% for bio-adhesives, though with substantial variability across specific formulations and system boundaries [40,42]. However, these climate benefits are frequently accompanied by increased burdens in other impact categories, particularly eutrophication associated with agricultural feedstock cultivation, which can increase substantially relative to fossil-based systems [42]. Similarly, bio-based and biodegradable sealant systems based on PLA or starch derivatives present challenges related to land use, water consumption, and the infrastructure requirements for industrial composting that are not always captured in simplified environmental claims [98]. These findings underscore that the environmental case for bio-based interfacial materials in packaging is system-dependent and cannot be reduced to a simple substitution narrative: full LCA accounting across multiple impact categories, including land use, water use, and end-of-life scenario assumptions, is necessary to support evidence-based material selection in circular packaging design.

Despite their conceptual appeal, debondable and stimuli-responsive adhesives face substantial barriers that currently limit their transition from laboratory demonstration to industrial packaging applications. A primary challenge is the fundamental trade-off between adhesion strength and reversibility: formulations designed to debond on demand must simultaneously provide sufficient interfacial strength under service conditions while retaining the capacity for clean, controlled separation at end of life. This functional trade-off represents the central engineering challenge in the field, and has not yet been resolved at performance levels compatible with high-speed packaging converting lines. The adoption of debondable adhesives is further constrained by synthetic challenges that limit practicality and scalability, as most responsive systems rely on specialised chemistries or encapsulation strategies difficult to reproduce at industrial volumes and competitive costs [99].

Beyond formulation complexity, the integration of stimulus-delivery systems into existing converting infrastructure represents a significant practical obstacle. Trigger conditions—whether thermal, photochemical, or chemical—must be compatible with line speeds, substrate variability, and process tolerances typical of industrial packaging environments, requirements that laboratory-scale demonstrations rarely address. While dismantlable adhesives capable of debonding on demand offer a promising paradigm for zero-waste industrial production, translating this potential into mainstream packaging applications requires overcoming substantial gaps between proof-of-concept performance and the robustness demanded by large-scale converting operations. Life-cycle benefits, though potentially significant, also remain largely unvalidated at scale, limiting the business case for industrial investment in these technologies [28].

### 6.5. Regulatory Drivers and Design Implications

Regulatory developments are increasingly shaping the evolution of adhesive and sealant technologies in packaging, system design, and sustainability strategies. Rather than acting solely as external constraints, regulations related to food safety, environmental protection, occupational exposure, and circular economy objectives now directly influence formulation chemistry, converting technologies, and packaging architecture.

In food-contact applications, regulatory requirements concerning migration safety, chemical transparency, and traceability have stimulated the development of low-migration adhesive formulations, improved curing chemistries, and more advanced analytical screening methodologies. Particular attention has been devoted to NIAS, residual monomers, and reaction by-products potentially migrating across packaging layers. The absence of harmonised EU-specific legislation for adhesives, highlighted in policy analyses of food-contact materials, continues to generate heterogeneous compliance approaches and practical challenges for enforcement and innovation [90].

Environmental and occupational safety regulations have similarly accelerated the transition toward solvent-free, water-based, and hot-melt adhesive technologies. The growing emphasis on reducing volatile organic compound emissions, hazardous substance exposure, and environmental impact has promoted the development of low-emission adhesive systems and alternative raw materials, including bio-based feedstocks derived from renewable resources such as vegetable oils and natural polymers [88]. These developments illustrate how regulatory pressure can simultaneously improve safety performance while fostering technological innovation.

Circular economy policies now exert an increasingly direct influence on adhesive design. Packaging recyclability targets, eco-design requirements, and environmental labelling initiatives are progressively integrating criteria related to material compatibility, recyclate quality, and life-cycle impact. Policy analyses emphasise that packaging requirements may constrain the adoption of certain materials or technologies when recyclability, recycled-content targets, or safety standards must be simultaneously satisfied [10,30].

**Figure 20:**
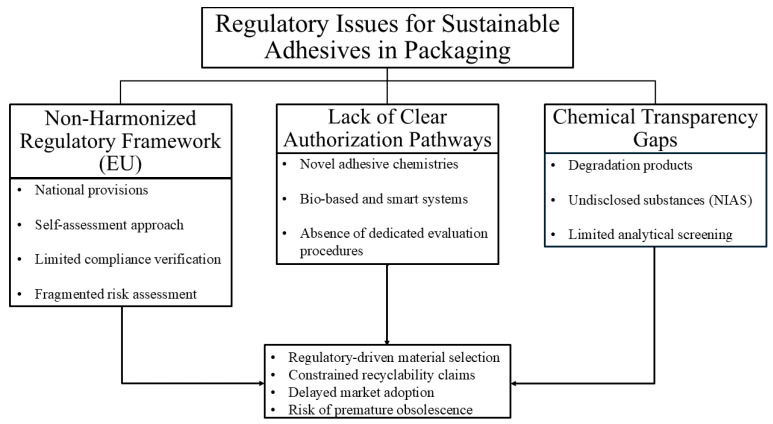
Regulatory challenges and design implications for sustainable adhesives in packaging. Conceptual overview of the main regulatory barriers affecting the development and adoption of sustainable packaging adhesives, including the lack of harmonised EU regulatory frameworks, unclear authorisation pathways for novel adhesive chemistries, and chemical transparency limitations such as NIAS identification. These factors increasingly influence material selection, recyclability claims, innovation uptake, and circular packaging design strategies.

At the same time, regulatory uncertainty remains a significant barrier to innovation. This fragmented regulatory landscape may slow the market introduction of emerging technologies such as bio-based adhesives, reversible bonding systems, or smart adhesive formulations, particularly where clear evaluation procedures for novel chemistries or degradation products are lacking [93] (Figure 20).

From a design perspective, these evolving regulatory pressures promote a shift from reactive compliance toward proactive regulatory-informed design. Packaging engineers and materials scientists increasingly integrate regulatory foresight into early development stages, considering safety, sustainability, recyclability, and functional performance concurrently rather than sequentially [81].

Looking forward, several strategic directions can be identified:

- increased harmonization of regulatory frameworks for packaging adhesives and sealants;
- improved analytical methods for migration safety and chemical transparency;
- development of adhesive systems compatible with circular economy targets;
- greater integration of life-cycle assessment and eco-design criteria in packaging development.

In the EU context, the main regulatory barriers for bioadhesives arise from the absence of a harmonised positive list specifically for packaging adhesives, the case-by-case safety demonstration required under Regulation (EC) No. 1935/2004, and the emerging recyclability and design-for-recycling requirements introduced by the PPWR.

## 7. Conclusions

Adhesives and sealants emerge from this review as structural enablers and system-level constraints within modern packaging architectures, whose influence extends across mechanical integrity, barrier continuity, process reliability, regulatory compliance, and end-of-life performance. The three-dimensional classification framework proposed here—integrating functional role, material chemistry and activation mechanism, and performance constraints—demonstrates that adhesive and sealant selection cannot be decoupled from packaging architecture, processing route, and regulatory context. A recurring finding across all material families is the fundamental tension between interfacial performance and circularity: chemically complex systems that deliver high bond or seal strength, broad processing windows, and thermal resistance tend to conflict with recyclability and migration safety requirements, while bio-based and biodegradable alternatives offer improved end-of-life compatibility at the cost of narrower processing tolerances and reduced functional robustness. Debondable and reversible interfacial systems represent a promising but not yet industrially mature pathway to reconcile these competing requirements.

The regulatory landscape surrounding packaging adhesives and sealants remains fragmented and incomplete, particularly with regard to food-contact compliance, NIAS identification, and harmonised end-of-life criteria.

This fragmentation currently constitutes a tangible barrier to the adoption of emerging solutions—including bio-based adhesives, debondable interfaces, and low-migration reactive systems—and introduces uncertainty into material innovation cycles. Addressing this gap will require coordinated progress between regulatory bodies, industry, and the research community, and represents one of the most practically significant non-technical challenges facing the field.

Several research priorities emerge directly from the limitations identified in this review. The performance gap between bio-based and synthetic systems under industrially relevant converting conditions remains insufficiently characterised, and establishing realistic thresholds for bond strength, seal initiation temperature, and hot-tack in bio-based formulations is a prerequisite for evidence-based substitution strategies in flexible and multilayer packaging. The industrial integration of debondable and stimuli-responsive adhesive systems requires validation across converting speeds, substrate combinations, and migration safety requirements before these technologies can displace conventional lamination adhesives at scale. The heterogeneity of test protocols for seal strength, SIT, and hot-tack across the literature—spanning ASTM F88, DIN 55529, and institution-specific methods—currently limits cross-study comparability and represents a methodological priority for the field. Finally, life-cycle assessment frameworks need to be extended beyond single-material metrics to capture architecture-level trade-offs between interfacial performance, recyclability, and chemical safety in multilayer packaging systems, providing a more complete basis for sustainable material selection and circular packaging design.

## Figures and Tables

**Table 1:** Comparative performance metrics for packaging adhesive systems (qualitative trends).

	Bond Integrity *	Converting Speed/Line Integration	Substrate Versatility	**Thermal & Environmental** Durability	End-of-Life Compatibility **
A. Chemical origin					
Natural/bio-based adhesives	Low–Moderate	Moderate	Limited–Moderate	Low–Moderate	High
Synthetic adhesives (generic)	Moderate–High to High	Moderate	High	High	Low–Moderate
B. Activation mechanism/processing route					
Hot-melt adhesives	Moderate	High	Moderate–High	Moderate	Moderate
Pressure-sensitive adhesives (PSA)	Low–Moderate	Very high	Moderate	Low–Moderate	Low–Moderate
Reactive/laminating adhesives (e.g., PU)	High	Moderate	High	High	Low
Water- or solvent-based adhesives	Moderate	Moderate	Moderate	Moderate	Moderate
Heat-sealable/heat-activated adhesives	Moderate	Moderate–High	Moderate	Moderate	Moderate
C. Functional application domain					
Tie layers/compatibilising interlayers	Function-specific	Integrated	Very high (targeted)	High	Low
Special-function interfaces (removable, wash-off, debond-on-demand)	Application-specific	Variable	Variable	Variable	Potentially high

*Note: The levels in this table are expressed in relative terms (Low → High) to summarize packaging-relevant trends rather than absolute values. They are derived by cross-reading packaging handbooks and classification manuals plus packaging-focused literature that links adhesive families to bond performance, process integration, and end-of-life constraints, and they are used here as a system-level comparison consistent with the framework in Section 2 and Section 3.* * Bond integrity refers to the ability of the adhesive interface to maintain mechanical integrity in the intended packaging configuration (e.g., laminate integrity, label retention), typically discussed through peel/shear/tack-type outcomes (test-dependent). ** End-of-life compatibility indicates whether the adhesive class tends to facilitate or hinder recycling/composting pathways (e.g., wash-off/removability for labels, contamination risk, delamination feasibility), rather than “biodegradability” in isolation.

**Table 2:** Comparative performance metrics for packaging sealant systems (qualitative trends).

Sealant Class	SIT	Seal Strength	Hot-Tack	Sealing Window	Barrier Continuity	End-of-Life Compatibility
Polyolefin-based	Low	High	High	Wide	Moderate	High (mono-material potential)
Barrier-oriented multilayer sealants	Moderate	High	Moderate	Narrow–Moderate	High	Low–Moderate
Biodegradable sealants	High	Low–Moderate	Low	Narrow	Low–Moderate	High (compostable contexts)
Advanced/functional systems	Variable	Application-specific	Variable	Process-dependent	Variable	Variable

*Note: Values should therefore be interpreted as comparative functional trends rather than absolute material properties, since seal performance depends strongly on sealing conditions (temperature, pressure, dwell time), substrate combinations, and package architecture.*

**Table 3:** Integrated system-level comparison of the principal adhesive and sealant systems used in packaging, highlighting the main trade-offs between interfacial performance, process robustness, and circularity compatibility. The comparison is intended as a qualitative synthesis of Table 1 and Table 2 and does not represent a direct numerical bench-mark across studies.

System/Family	Interfacial Performance	Process Robustness	Circularity Compatibility	Main Trade-Off
Natural/bio-based adhesives	Moderate	Limited	High	Sustainability vs. moisture resistance
Synthetic adhesives	High	High	Limited	Performance vs. recyclability
Hot-melt systems	Moderate–High	High	Moderate	Fast processing vs. thermal resistance
Pressure-sensitive adhesives	Moderate	High	Variable	Adhesion stability vs. removability
Reactive/laminating adhesives	Very high	High	Poor	Strong interfacial bonding vs. difficult material separation
Conventional thermoplastic sealants	High	High	Moderate–High	Seal reliability vs. limited intrinsic barrier performance
Barrier-oriented multilayer sealants	Very high	Moderate–High	Low–Moderate	Barrier continuity vs. multimaterial complexity
Biodegradable/bio-based sealants	Moderate	Limited	High	Circularity vs. sealing robustness

## Data Availability

No new data were created or analyzed in this study. Data sharing is not applicable to this article.
